# Enhancing the Dispersibility and Stability of Graphene in Water Using Porphyrin‐Based Compounds

**DOI:** 10.1002/smtd.202401431

**Published:** 2025-03-10

**Authors:** Katerina Anagnostou, Evangelos Sotiropoulos, Nikolaos Tzoganakis, Christos Polyzoidis, Konstantinos Rogdakis, Anna Katsari, Katerina Achilleos, Evitina Triantafyllou, Georgios Landrou, Emmanouil Nikoloudakis, Georgios Charalambidis, Athanassios G. Coutsolelos, Emmanuel Kymakis

**Affiliations:** ^1^ Department of Electrical & Computer Engineering Hellenic Mediterranean University (HMU) Heraklion 71410 Greece; ^2^ Institute of Emerging Technologies (i‐EMERGE) of HMU Research Center Heraklion 71410 Greece; ^3^ Laboratory of Bioinorganic Chemistry Chemistry Department University of Crete Heraklion 70013 Greece; ^4^ Theoretical and Physical Chemistry Institute National Hellenic Research Foundation 48 Vassileos Constantinou Avenue Athens 11635 Greece; ^5^ Institute of Electronic Structure and Laser (IESL) Foundation for Research and Technology – Hellas (FORTH) Vassilika Vouton Heraklion 70013 Greece

**Keywords:** 2D nanomaterials, dispersibility, dispersion stability, electrochemically exfoliated graphene, graphene, metalloporphyrins, porphyrins

## Abstract

Although graphene's superior electrical, optoelectronic, thermal, and mechanical properties have been evident for 20 years now, its poor water dispersibility has hindered its incorporation in many types of applications and technologies. Strong examples of this are biomedical and environmental applications and devices that require non‐toxic, biocompatible media and not toxic organic solvents like N‐N’‐Dimethylformamide, in which graphene is readily dispersible. In this work, we investigate a new way to prepare high‐concentration and stable graphene dispersions in water by employing porphyrin‐based compounds as stabilisers. To this end, electrochemically exfoliated graphene (EEG) and assess the potential of five porphyrins and metalloporphyrins are prepared to disperse EEG in water successfully. The dispersibility and stability of EEG in each porphyrin aqueous solution are evaluated by recording their UV–vis absorption spectra. Two of the synthesised compounds, namely sodium salt of 5,10,15,20‐tetrakis(4‐carboxyphenyl)‐porphyrin or TCPP and sodium salt of [5,10,15,20‐tetrakis(4‐carboxyphenyl)‐porphyrinato]tin(IV) or Sn‐TCPP , are successful in stably dispersing EEG in water. The intermolecular interaction between the EEG flakes and [H_2_TCPP]Na_4_ and [Sn(OH)_2_TCPP]Na_4_ molecules are investigated via fluorescence emission spectroscopy. Finally, solid thin films of the EEG(TCPP) and EEG(Sn‐TCPP) dispersions are prepared via spray‐coating, and their optoelectronic properties and surface morphology are investigated.

## Graphical abstract

A new method is investigated for the preparation of high‐concentration and stable graphene dispersions in water; by employing porphyrin‐based compounds as stabilizers for the dispersion of Electrochemically Exfoliated Graphene (EEG).

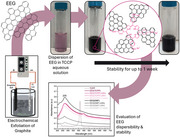

## 1 Introduction

Since its isolation in 2004, graphene has established itself as a promising material in the field of nanotechnology by virtue of its exceptional optoelectronic, thermal, and mechanical properties.^[^ ^1^ ^]^ This carbon allotrope has been studied extensively over the past 20 years and has already found application in emerging photovoltaics, electronic computer components, such as transistors, third‐generation batteries, biosensors and gas sensors, water filtration systems, within the biomedical field in advanced drug delivery systems, even in space application technologies.^[^ ^2^, ^3^, ^4^, ^5^, ^6^, ^7^, ^8^, ^9^, ^10^ ^]^ The mechanical and optoelectronic properties that make graphene so versatile in nanotechnology applications derive from its chemical and electronic structure. Graphene consists solely of carbon (C) atoms linked with strong interchanging single (C─C) and double (C═C) covalent bonds arranged in a hexagonal lattice, forming a 2D aromatic structure that is only one atom thick. Each atom is sp^2^ hybridised, forming three strong σ bonds with three neighbouring atoms, resulting in a planar structure. The π‐electrons from the p‐orbital form a conjugated π‐system across the benzene‐ring lattice. While the σ‐bonds and tightly packed carbon atoms are responsible for graphene's enhanced strength, and thermal stability^[^ ^11^, ^12^, ^13^ ^]^ the π‐bonds formed from the overlapping vertical p‐orbitals, allow free movement of the π‐electrons, resulting in graphene's exceptional electron mobility and high conductivity.^[^ ^12^, ^14^, ^15^ ^]^ Other valuable properties include flexibility and high transparency due to atomic thickness.^[^ ^16^, ^17^, ^18^ ^]^ Even few‐layer graphene exhibits remarkable electrical and optoelectronic properties.^[^ ^1^, ^19^ ^]^

To take advantage of graphene's properties within emerging large‐scale devices and technologies, it is much more efficient for this material to be prepared in a ready‐to‐deposit, liquid‐processable form that is compatible with printing methods, i.e., a dispersion, composite, ink, or paste.^[^ ^20^, ^21^, ^22^ ^]^ The preparation of 2D and few‐layer nanomaterials through liquid processes allows for high‐yield ink production, as opposed to bottom‐up methods, such as Chemical Vapor Deposition (CVD), which produces high‐quality graphene but is costly, yields limited amounts on small‐area substrates, and requires a sensitive transfer process of the materials from the metal substrate.^[^ ^23^, ^24^ ^]^ When considering the liquid‐processability of graphene, one limitation is immediately evident. Because graphene is composed of only carbon atoms and lacks any functional groups, it is highly hydrophobic, which severely limits its dispersibility in most common non‐toxic, green, and low‐cost solvents, such as water and alcohol. Even though high‐concentration graphene dispersions can be achieved in organic solvents such as N,N‐Dimethylformamide (DMF), Dimethyl sulfoxide (DMSO), and N‐Methyl‐2‐Pyrrolidone (NMP), achieving stable dispersions in water is significantly important.^[^ ^25^, ^26^, ^27^ ^]^ First, when incorporating materials into biomedical and environmental applications, the reagents and liquid media involved should be biocompatible, non‐toxic, and eco‐friendly, making the use of toxic solvents undesirable in these cases. Additionally, organic solvents like the previously mentioned DMF and NMP are not only highly hazardous and not ecological but also have very high boiling points, making them difficult to remove during the deposition of graphene inks. Furthermore, water is safe, naturally abundant, and inexpensive, making it an ideal medium for scalable material deposition processes. For these reasons, achieving graphene dispersions in water‐based media is a critical challenge in harnessing this material's full potential in a wide array of large‐area devices and applications.

Various methods have been employed in past research to improve the water dispersibility of graphene. For instance, a common method is to chemically modify graphene's structure by introducing oxygen functional groups into the graphitic lattice via oxidation, to form graphene oxide (GO).^[^ ^28^, ^29^, ^30^, ^31^, ^32^ ^]^ In this case, although the oxygen‐containing groups significantly boost the material's hydrophilicity and water dispersibility, their presence also disrupts the sp^2^ hybridised system of the graphene lattice and, as a result, minimises the electron mobility and conductivity, making GO an insulator.^[^ ^29^ ^]^ The electronic properties can be partially recovered through the partial removal of some of the oxygen groups via chemical, thermal or laser reduction to produce reduced graphene oxide (RGO).^[^ ^31^, ^33^, ^34^, ^35^, ^36^ ^]^ The result in this case is an intermediate structure in which the sp^2^ hybridised system and, subsequently, the conductivity, are partially restored, while the oxygen groups that remain help RGO maintain some water dispersibility. This sequence of processes, however, is time and resource‐consuming and does not result in pure graphene, but rather, an oxygenated graphene derivative. An alternative method is to chemically functionalise graphene by covalently linking hydrophilic polymers to its surface.^[^ ^37^ ^]^ This approach also fundamentally alters graphene's chemical makeup and, by extent, its inherent properties.^[^ ^38^ ^]^ Surfactant‐assisted stabilization has also been employed in past research as a way to enhance the dispersibility of graphene in water without intervening in its chemical makeup.^[^ ^39^, ^40^, ^41^ ^]^ **Table** **1**. provides a brief literature review on different surfactants that have been previously investigated as a means of aqueous graphene dispersion with the respective stability times and concentrations.

**Table 1:** Surfactant Stability and Concentration Data for Aqueous Graphene Dispersions.

Surfactant	Stability Time	Concentration	Refs.
PVD (Polyvinylidene)	1 week	0.1 mg ml^−1^	[39]
PVA (Polyvinyl Alcohol)	N/A	2 mg ml^−1^	[40]
CTAB (Cetyltrimethylammonium Bromide)	200 h	0.4 mg ml^−1^	[41]
SDS (Sodium Dodecyl Sulfate)	Up to 72h	2.5 mg ml^−1^	[42]
SDBS (Sodium Dodecylbenzenesulfonate)	22 days	10 mg ml^−1^	[43]
Triton X‐100 (Polyethylene Glycol tert‐Octylphenyl Ether)	up to 60 min	1 mg ml^−1^	[44]
BPS (Bisphenol S)	Several months	0.78 mg ml^−1^	[45]
Py‐SASS (Pyrene‐Sodium Alkyl Sulfonate Surfactant)	Several months	1 mg ml^−1^	[46]
SDBS (Sodium Dodecylbenzenesulfonate)	N/A	0.22 mg ml^−1^	[46]
PVP (Polyvinylpyrrolidone)	several weeks	1 mg ml^−1^	[46]
SC (Sodium Cholate)	Up to 5 days	0.3 mg ml^−1^	[47]

In this study, we aim to achieve high‐concentration and stable graphene dispersions in water without chemically modifying its structure, but rather with the assistance of hydrophilic porphyrin‐based additives that are able to interact non‐covalently with the graphene flakes. We elected to produce the graphene used for this investigation via electrochemical exfoliation of pure graphite, as it is one of the most economical, time‐saving, and tuneable one‐step techniques for graphene nanosheet production.^[^ ^25^, ^48^, ^49^ ^]^ During electrochemical exfoliation, the applied voltage drives the ions produced from the electrolyte to intercalate, i.e., insert themselves, between the layers of the bulk graphite electrode. This ion intercalation causes the layers to expand and separate leading to exfoliation. In this work, cathodic exfoliation was employed as a technique for functional‐grade graphene production, wherein the graphite source acts as a cathode and is intercalated with cations generated from the electrolyte.^[^ ^50^ ^]^ Electrochemically exfoliated graphene (EEG) was produced using graphite foil as the cathode and platinum (Pt) foil as the anode in aqueous ammonium sulfate (NH_4_)_2_SO_4_ electrolyte solution. The resulting EEG was characterised using Attenuated Total Reflectance IR (ATR‐IR) and Raman spectroscopy, as well as X‐ray Diffraction (XRD), to evaluate its purity and ensure that no oxidation took place during electrochemical exfoliation.

The produced EEG was dispersed in aqueous solutions of five different porphyrin‐based compounds, to assess their ability to successfully and stably disperse graphene in water. Porphyrins are macromolecular heterocyclic organic derivatives with various substituents at the periphery of the porphyrin ring. Owing to the extended π‐aromatic system, porphyrins exhibit excellent thermal and chemical stability and unique photophysical and electrochemical properties. Various metal ions can be coordinated in the porphyrin centre to form metal complexes, also known as metalloporphyrins. The most well‐known natural porphyrins are heme (being responsible for oxygen transport in the blood stream), chlorophyll (an essential component in photosynthesis), and bacteriochlorophyll (accountable for photosynthesis in bacteria). Numerous synthetic porphyrins have been covalently connected with 2D materials such as graphene oxide and MoS_2_, enhancing the dispersibility of the materials in various solvents.^[^ ^51^, ^52^ ^]^ In this work, we explored the potential of:

- Sodium salt of 5,10,15,20‐tetrakis(4‐carboxyphenyl)‐porphyrin or [H_2_TCPP]Na_4_
- Sodium salt of [5,10,15,20‐tetrakis(4‐carboxyphenyl)‐porphyrinato]tin(IV) or [Sn(OH)_2_TCPP]Na_4_
- 5,10,15,20‐tetrakis‐(N‐methyl‐4‐pyridinium)‐porphyrin iodide or [H_2_TMPyP]I_4_)
- [5,10,15,20‐tetrakis‐(N‐methyl‐4‐pyridinium)‐porphyrinato]tin(IV) chloride or [SnCl_2_TMPyP]Cl_4_
- [5,10,15,20‐tetrakis‐(4‐pyridinium)‐porphyrinato]tin(IV) or Sn(OH)_2_TMPyP

**Figure 1:**
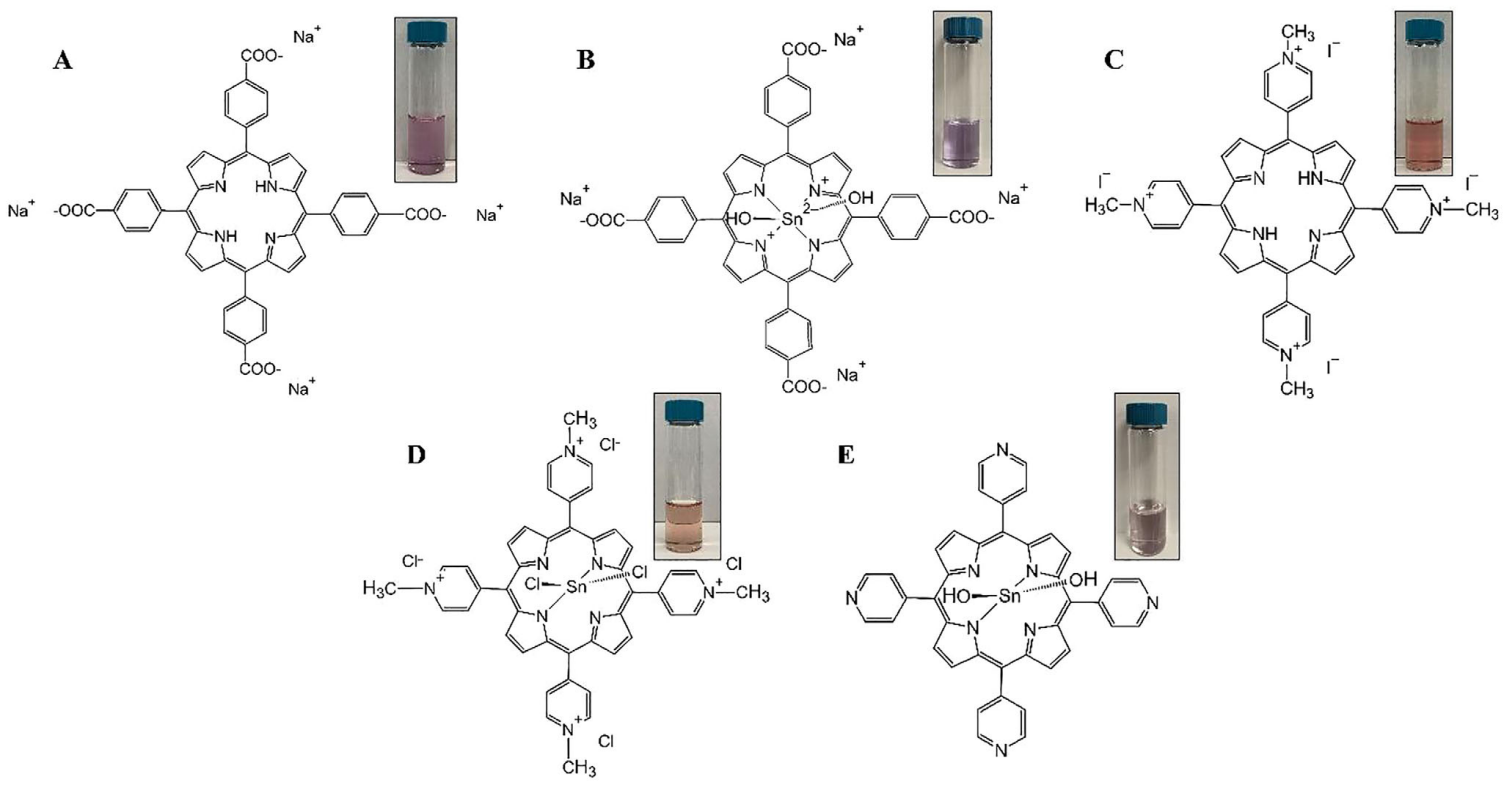
Chemical structures of porphyrin‐based compounds tested for their potential to produce stable EEG dispersions in water. A) [H_2_TCPP]Na_4_, B) [Sn(OH)_2_TCPP]Na_4_, C) [H_2_TMPyP]I_4_, D) [SnCl_2_TMPyP]Cl_4_ and E) Sn(OH)_2_TMPyP. Digital photographs of each aqueous porphyrin solution (10^−5^ M) used are also included.

To act as surfactants for the formulation of stable EEG dispersion in water (**Figure** **1**).

## 2 Results and Discussion

### 2.1 Characterization of EEG and Porphyrin Compounds

To evaluate the chemical structure and quality of the produced EEG, ATR‐IR, and Raman spectroscopy, as well as XRD, were employed.^[^ ^53^, ^54^, ^55^, ^56^ ^]^ The ATR‐IR spectrum of EEG (Figure S1A, Supporting Information) displays a peak at 1587 cm^−1^ which is attributed to the C═C aromatic stretching vibrations.^[^ ^53^, ^54^ ^]^ Notably, no evident peaks attributed to C─O vibrations, typical of graphene oxide (GO), are detected, indicating that the produced EEG is not oxidised during the electrochemical exfoliation.^[^ ^31^, ^55^ ^]^

The Raman spectrum of graphite foil (Figure S1B, Supporting Information) exhibits the characteristic G and D bands at 1579.9 cm^−1^ and 1347.8 cm^−1^, respectively.^[^ ^57^ ^]^ The G band corresponds to the first‐order scattering of the doubly degenerate E_2_ _g_ phonon at the Brillouin zone centre and originates from the primary in‐plane vibrational mode of the sp^2^ hybridised carbon atoms. The D band derives from the breathing mode of A_1_ _g_ symmetry phonons around the K or K’ points of the Brillouin zone. The G peak for the graphite foil is very sharp and narrow, while the D peak is very weak, which aligns with previous reports.^[^ ^58^, ^59^ ^]^ The D peak is a disorder‐induced Raman mode and is associated with structural imperfections in the graphite flakes.^[^ ^59^, ^60^ ^]^ The I_D_/I_G_ ratio of graphite foil is calculated at 0.04. The asymmetrical peak at 2717.8 cm^−1^ is assigned to the 2D band, while the lower intensity peak at 3245.6 cm^−1^, is assigned to the 2D band, which are both second‐order Raman modes.^[^ ^61^, ^62^ ^]^ Two more low‐intensity peaks are observed at 2437.2 and 2992.7 cm^−1^, attributed to the D+D″, and the D+D’ Raman signals, respectively, and occur due to combination scattering.^[^ ^63^ ^]^ Shifting focus to the exfoliated graphene sample, the overall Raman spectrum of the as‐prepared EEG matches those previously reported for electrochemically exfoliated graphene samples.^[^ ^64^, ^65^, ^66^ ^]^ The D and G bands appear at 1353.5 and 1579.4 cm^−1^, respectively, with the intensity of the D band increasing to be higher than that of the graphite precursor, but still significantly lower than that of the G band, which is inagreement with Raman spectra in previous graphene studies.^[^ ^57^, ^67^, ^68^, ^69^ ^]^ The I_D_/I_G_ ratio for EEG increases to 0.34, which is within the range of values previously reported for graphene produced via electrochemical exfoliation.^[^ ^48^, ^70^, ^71^, ^72^, ^73^, ^74^, ^75^, ^76^ ^]^ The 2D and 2D’ bands, observed at 2715.6 and 3242.3 cm^−1^, respectively, correspond to two second‐order Raman scattering modes activated in pristine graphene.^[^ ^62^ ^]^ The 2D band becomes more symmetrical upon electrochemical exfoliation. The calculated I_2D_/I_G_ ratio for EEG increases to 0.30, which indicates the decrease in the number of graphene layers and agrees with previous reports on electrochemically exfoliated graphene.^[^ ^76^, ^77^, ^78^, ^79^, ^80^ ^]^ This I_2D_/I_G_ ratio is smaller than that of single‐layer graphene, but is, however, indicative of multi‐layer graphene.^[^ ^80^, ^81^, ^82^ ^]^ Lastly, The D+D″ and D+D’ combination Raman modes are also detected for EEG at 2458.5 and 2929.1 cm^−1^, respectively.

The recorded XRD patterns of the produced EEG powder and the graphite foil precursor are featured in Figure S2 (Supporting Information). The graphite foil sample exhibits a very sharp and narrow diffraction peak at 26.36°, which corresponds to the (002) Bragg's plane and is typical of the highly crystalline structure of graphite.^[^ ^83^, ^84^ ^]^ The angle of the graphite (002) peak corresponds to an interplanar spacing of d_002_ = 3.4724 Å according to Bragg's equation (Equation 1). A much lower intensity peak appears at 54.44°, which corresponds to the (004) orientation.^[^ ^85^, ^86^ ^]^ The diffraction peaks that appear at 38.17° and 44.41° (marked **) appear due to the XRD sample holder which is made of aluminium (Al) and are assigned to the (111) and (200) lattice planes of Al, respectively.^[^ ^87^, ^88^ ^]^

(1) $$d=\frac{n\lambda }{2\;sin\theta }\;$$

where:

*n = diffraction order*

*λ = XRD radiation wavelength (Å)*

*d = interplanar distance (Å)*

*θ = Bragg angle (^o^)*

In the EEG XRD pattern, the (004) peak disappears, which aligns with previously reported XRD patterns for graphene samples.^[^ ^89^, ^90^ ^]^ The (002) peak decreases in intensity and shifts to a slightly lower 2θ angle (26.31°), meaning the interplanar spacing increases to d_002_ = 3.4786 Å. The (002) also becomes broader, with the FWHM increasing from 0.29 for graphite foil to 0.81 for EEG. These observations are indicative of successful exfoliation of the graphite foil.^[^ ^84^, ^91^, ^92^, ^93^, ^94^ ^]^ No diffraction peak is detected at ≈11–13° in the EEG XRD pattern, which is additional confirmation that the produced graphene is not oxidised during the electrochemical exfoliation process.^[^ ^86^, ^95^, ^96^ ^]^ The average nanocrystallite size of EEG was calculated using the Scherrer equation (Equation 2) and found to be 10.46 nm, which is significantly smaller than that calculated for the graphite foil precursor, at 29.02 nm.^[^ ^97^ ^]^

(2) $$L=\frac{K\lambda }{\beta \cdot \cos\theta }$$

where:

*L = nanocrystallite size (nm)*

*K = dimensionless shape constant*

*λ = XRD radiation wavelength*

*β = full width half maximum (FWHM) of XRD peak*

*θ = Bragg angle (^o^)*

All porphyrin derivatives were prepared according to known experimental procedures.^[^ ^98^, ^99^, ^100^, ^101^ ^]^ The successful synthesis of the porphyrin and metalloporphyrin compounds was verified via ^1^H‐NMR (Figure S3–S6, Supporting Information) and MALDI‐TOF spectroscopy (Figure S7, Supporting Information). The ^1^H‐NMR spectrum of [Sn(ΟH)_2_TCPP]Na_4_ could not be obtained due to solubility issues, therefore its successful preparation was confirmed from the MALDI‐TOF mass spectrum.

### 2.2 Dispersibility and Stability of EEG in Porphyrin Aqueous Solutions

EEG was successfully dispersed in [H_2_TCPP]Na_4_ (Figure S8A, Supporting Information) and [Sn(OH)_2_TCPP]Na_4_ (Figure S8B, Supporting Information), both of which showed superior stability for up to a week. [SnCl_2_TMPyP]Cl_4_ yielded a low concentration EEG dispersion with poor stability, as the graphene flakes precipitated within 24 h (Figure S8C, Supporting Information). Lastly, [H_2_TMPyP]I_4_ (Figure S8D, Supporting Information) and Sn(OH)_2_TMPyP (Figure S8E, Supporting Information) did not disperse EEG. Interestingly, the supernatants that are collected from the [H_2_TMPyP]I_4_, [SnCl_2_TMPyP]Cl_4,_ and Sn(OH)_2_TMPyP are transparent, no longer exhibiting the colour of the respective porphyrins. EEG (1 mg ml^−1^) was dispersed in DMF following the same ultrasonication and centrifugation parameters to use as a reference (Figure S8F, Supporting Information).

**Figure 2:**
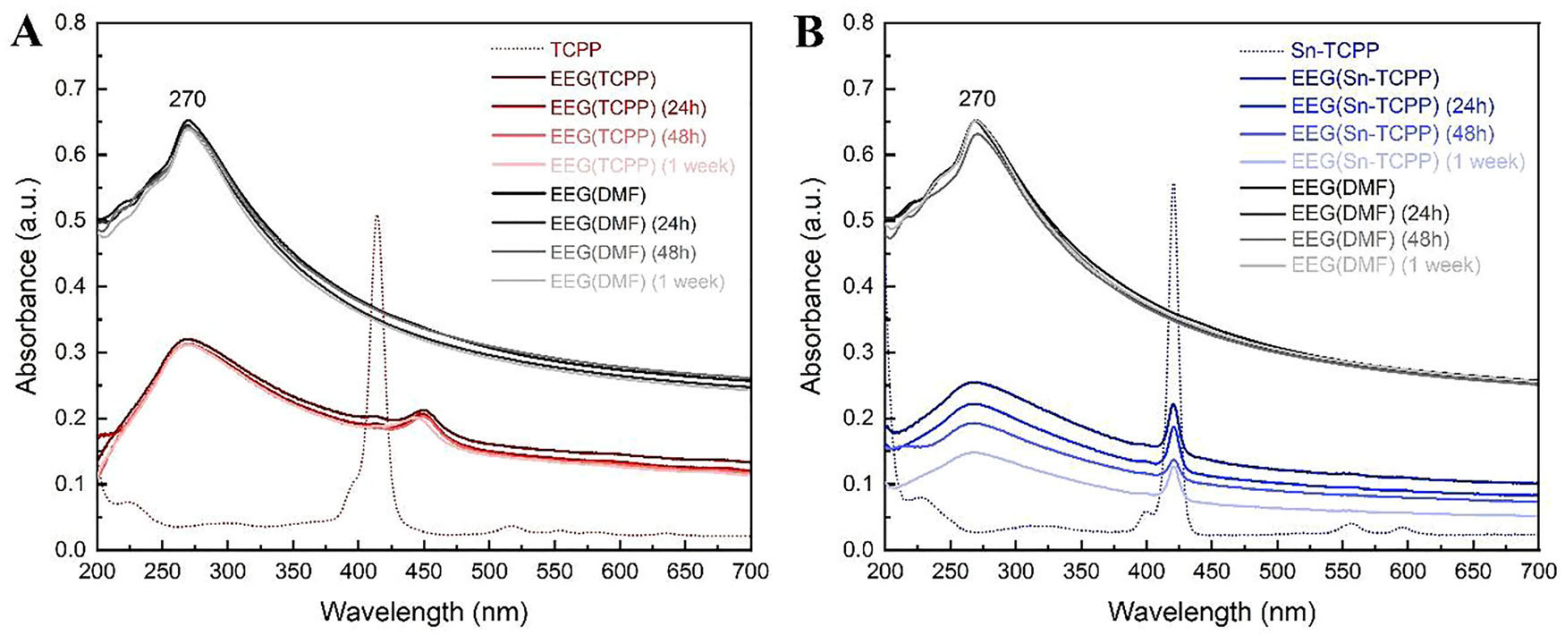
UV–vis absorption spectra of EEG (1 mg ml^−1^) dispersed in: A) TCPP and (B) Sn‐TCPP, recorded immediately after preparation, after 24 h, after 48 h, and after 1 week. Both graphs display the respective absorption spectra recorded for EEG(DMF) (1 mg ml^−1^) for reference.

**Figure 3:**
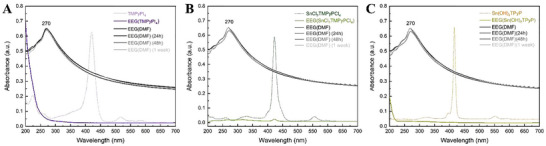
UV–vis absorption spectra of EEG (1 mg ml^−1^) dispersed in: A) [H_2_TMPyP]I_4_, B) [SnCl_2_TMPyP]Cl_4_ and C) Sn(OH)_2_TMPyP recorded immediately after centrifugation (EEG samples not diluted). The obtained spectra display neither the characteristic graphene peak nor the characteristic porphyrin peaks.

The UV–vis absorption spectra of EEG (1 mg ml^−1^) dispersed in the five porphyrin aqueous solutions were recorded and compared to that of EEG(DMF) as a reference (**Figures** **2** and **3**). Since [H_2_TMPyP]I_4_, [SnCl_2_TMPyP]Cl_4_, and Sn(OH)_2_TMPyP displayed no EEG dispersion stability, the UV–vis absorption spectra at the 24 h, 48 h, and 1‐week timepoints were not recorded for these samples. Conversely, as [H_2_TCPP]Na_4_ and [Sn(OH)_2_TCPP]Na_4_ displayed adequate EEG dispersibility and noteworthy stability, this work shifts its focus toward these two porphyrins, which will from now on be referred to as TCPP and Sn‐TCPP, respectively.

The characteristic maximum absorption peak of graphene at ≈270 nm, which is attributed to π → π* transitions of the aromatic C─C bonds, is clearly visible in the spectra of the EEG(DMF) reference and the EEG(TCPP), EEG(Sn‐TCPP) samples (Figure 2).^[^ ^102^, ^103^ ^]^ The position of the EEG peak is an indication of its purity, as the oxidation of graphene causes the absorption peak to shift to lower wavenumbers.^[^ ^104^, ^105^ ^]^ The absence of the characteristic shoulder that appears at ≈300 nm for graphene oxide due to n→ π* transitions of oxygen functional groups, is an additional indication of the purity of EEG.^[^ ^31^, ^106^ ^]^ The porphyrin and metalloporphyrin reference spectra exhibit a characteristic strong absorption signal at ≈420 nm (Soret band) and lower intensity peaks in the region of ≈515–595 nm (Q‐bands).^[^ ^107^, ^108^, ^109^ ^]^ The TCPP Soret band is still visible in the EEG(TCPP) spectra (Figure 2A), however it has red‐shifted to ≈447 nm. This phenomenon occurs as a result of the non‐covalent π‐π interactions between the EEG flakes and the porphyrin ring within TCPP's structure.^[^ ^110^, ^111^ ^]^ Similarly, the Soret band of Sn‐TCPP is also detected within the spectra of the EEG(Sn‐TCPP) samples, however little to no red‐shifting is observed in this case.

TCPP and Sn‐TCPP successfully dispersed the graphene flakes in water, albeit with lower concentrations than DMF. Notably, TCPP exhibits superior dispersion stability, as the absorbance intensity of graphene remains virtually the same even after 1 week. This stability behaviour in TCPP rivals that of EEG dispersed in DMF. Sn‐TCPP on the other hand, produces a lower‐stability dispersion, as the EEG absorption intensity is reduced by half after 1 week.

We theorize that the aqueous solutions of TCPP and Sn‐TCPP disperse the EEG flakes by the following intermolecular interaction mechanism: The porphyrin ring and the four phenyl rings within the chemical structure of these two compounds have the ability to form non‐covalent π‐π interactions with the aromatic lattice of the EEG flakes.^[^ ^112^, ^113^, ^114^ ^]^ Because both porphyrin compounds also have four carboxyl groups within their structure, we speculate that while the EEG lattice attaches via π‐π interactions, the highly hydrophilic carboxyl groups simultaneously interact with the surrounding water molecules, thus keeping the EEG flakes suspended. The other three porphyrin compounds lack carboxyl groups and are unable to successfully disperse the EEG flakes. This observation leads to the conclusion that the ─COOH groups are crucial for this graphene dispersion mechanism.

**Figure 4:**
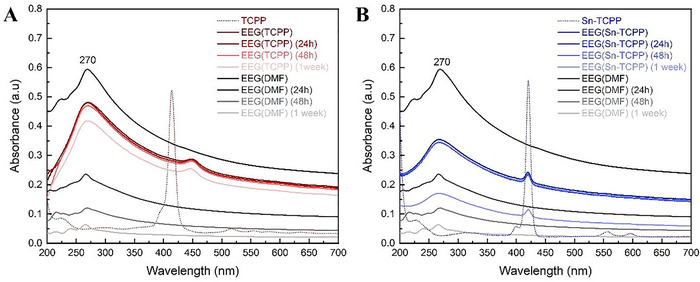
UV–vis absorption spectra of EEG (2.5 mg ml^−1^ in water) dispersed in: A) TCPP; B) Sn‐TCPP, recorded immediately after preparation, after 24 h, after 48 h, and after 1 week. Both graphs display the respective absorption spectra recorded for EEG(DMF) (2.5 mg ml^−1^) for comparison of EEG dispersibility and stability.

After establishing the ability of TCPP and Sn‐TCPP to stably disperse EEG flakes in water, a higher concentration was attempted. The initial EEG concentration was increased to 2.5 mg ml^−1^, keeping the same ultrasonication and centrifugation parameters, and the UV–vis absorption measurements were repeated (**Figure** **4**). When studying the resulting UV–vis absorption spectra of EEG(TCPP)(2.5 mg ml^−1^) and EEG(Sn‐TCPP)(2.5 mg ml^−1^), it is evident that both porphyrins are able to disperse higher amounts of EEG than the initially tested concentration of 1 mg ml^−1^. TCPP still achieves higher EEG dispersibility than Sn‐TCPP, although the EEG concentration is still lower than that achieved in DMF. However, both TCPP and Sn‐TCPP surpass DMF in terms of stability, with both samples exhibiting a decrease in absorption intensity only at the 1‐week mark. EEG(DMF) (2.5 mg ml^−1^) on the other hand begins to lose its dispersion stability even 24 h after preparation.

**Figure 5:**
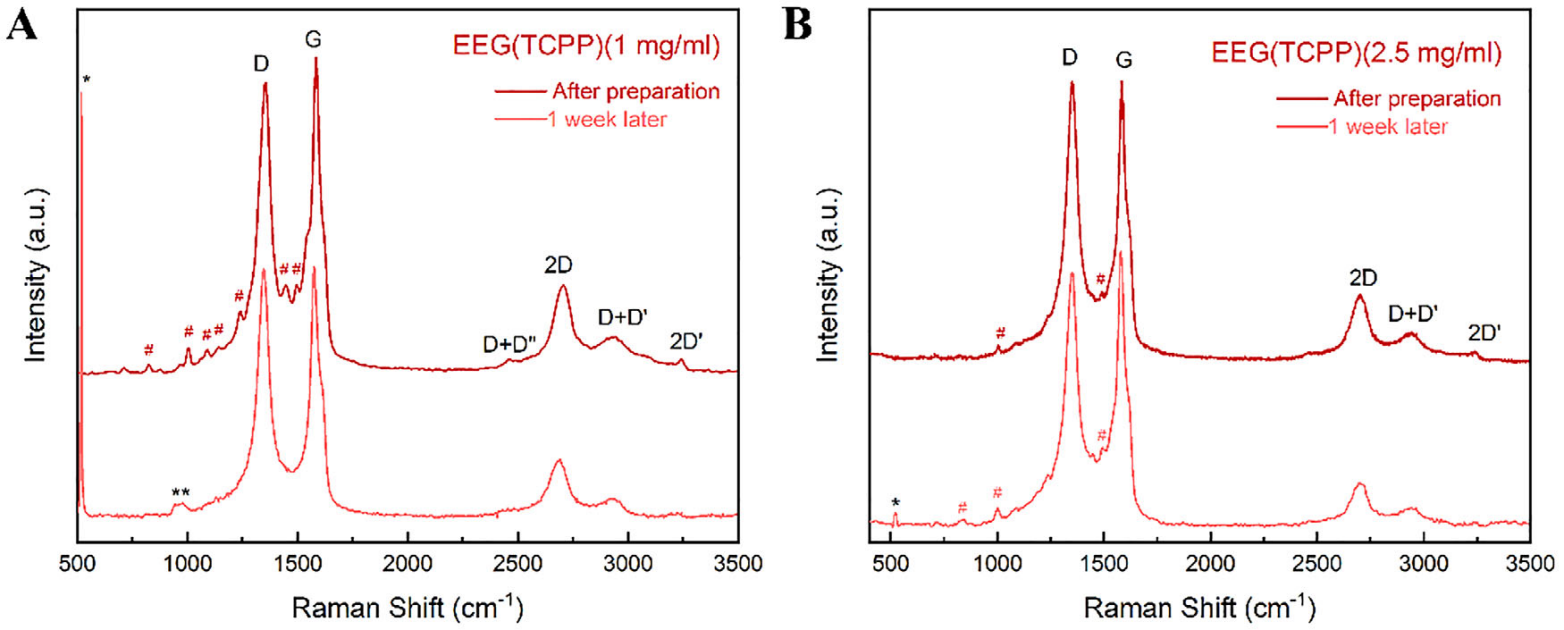
Raman spectra of A) EEG(TCCP) (1 mg ml^−1^) and B) EEG(TCPP) (2.5 mg ml^−1^) immediately after dispersion and 1 week after preparation.

The successfully and stably dispersed EEG samples were further characterised using Raman and ATR‐IR spectroscopy, as well as Scanning Electron Microscopy (SEM) and Transmission Electron Microscopy (TEM) imaging, to investigate their purity, structure, and morphology both immediately after preparation and after 1 week. Raman samples were prepared by drop‐casting each sample on O_2_‐plasma‐treated silicon (Si) wafer substrates and dried at room temperature. The resulting Raman spectra are featured in **Figures** **5** **–** **7**. The peaks that appear at 518 cm^−1^ (marked *) and 980 cm^−1^ (marked **) are attributed to the Si substrates.^[^ ^115^ ^]^ The low‐intensity peaks detected in the EEG(TCPP) spectra between ≈700–1500 cm^−1^ are assigned to Raman modes of TCPP.^[^ ^116^, ^117^, ^118^ ^]^ For all EEG samples, the I_D_/I_G_ increases after the one‐week stability test, as seen in **Table** **2**, pointing to a higher level of disorder in the EEG flakes that remain suspended after 1 week. Conversely, the I_2D_/I_G_ ratio decreases, showing that after 1 week, the larger EEG flakes consisting of more graphene layers precipitate, leaving the fewer‐layer EEG flakes dispersed.

**Table 2:** I_D_/I_G_ and I_2D_/I_G_ ratios were calculated from the Raman spectra of all EEG samples recorded immediately after dispersion preparation and at the 1‐week timestamp.

Sample	Timestamp	I_D_/I_G_	I_2D_/I_G_
EEG(TCPP) (1 mg ml^−1^)	0 h	0.93	0.29
	1 week	0.99	0.24
EEG(TCPP) (2.5 mg ml^−1^)	0 h	0.74	0.25
	1 week	0.81	0.23
EEG(SnTCPP) (1 mg ml^−1^)	0 h	1.13	0.19
	1 week	1.23	0.18
EEG(Sn‐TCPP) (2.5 mg ml^−1^)	0 h	1.16	0.17
	1 week	1.32	0.14
EEG(DMF) (1 mg ml^−1^)	0 h	0.98	0.22
	1 week	1.07	0.20
EEG(DMF) (2.5 mg ml^−1^)	0 h	1.12	0.26
	1 week	1.32	0.22

The ATR‐IR spectra of all EEG flake samples were also obtained with results presented in **Figure** **8**. Two strong peaks appear for all the spectra at ≈760 cm^−1^ and ≈895 cm^−1^, both of which are attributed to the aromatic C─H bending vibrations.^[^ ^119^, ^120^, ^121^ ^]^ These peaks arise and become more prominent than the C═C stretching peak (≈1600 cm^−1^) after dispersing EEG in the three different media. The C═C stretching peak is also detected at ≈1570 cm^−1^ at a lower intensity. Characteristic ATR‐IR peaks attributed to C─O bonds are not detected, showing that no oxygen impurities are present and, therefore, no oxidation of the EEG samples.^[^ ^122^ ^]^ The results agree with the previously analysed UV–vis absorption spectroscopy results, in that the intensity of the characteristic peaks decreased for the 1‐week samples.

SEM was used to gain insight into the morphology and structure of the dispersed EEG flakes. Samples for SEM were prepared by diluting the 2.5 mg ml^−1^ samples 100 times and drop‐casting 20 µl onto Si wafers that were mildly heated at 40 °C to evaporate the solvent. The Si substrates are first treated with O_2_‐plasma (50 W, 5 min). The resulting SEM images are presented in **Figure** **9**. Observing the x10000 SEM images, the TCPP stabiliser appears to facilitate smaller EEG flakes which are not as densely packed (Figure 9A). For EEG(DMF) (Figure 9C), the graphene flakes cover a larger area, perhaps due to the slower evaporation of the DMF solvent, which results in a larger, more compact assembly of graphene flakes. The EEG(Sn‐TCPP) sample (Figure 9B) exhibited intermediate levels of EEG flake aggregation. Shifting the focus to the x50000 SEM images (Figure 9D–F), it is evident that TCPP, similarly to DMF, yields more well‐defined EEG flakes than Sn‐TCPP does.

Lastly, TEM was employed to gain further insight into the structure and number of layers of the produced EEG flakes. To prepare samples for TEM, the 2.5 mg ml^−1^ dispersions were diluted 100 times, and the EEG flakes were deposited onto copper (Cu) TEM substrates by dip‐coating and subsequent drying at room temperature. The obtained images for the EEG(TCPP), EEG(Sn‐TCPP), and EEG(DMF) samples are shown in **Figures** 10, 11, 12, respectively, further confirming the production of exfoliated graphite. The EEG(TCPP) samples showed graphene flakes with a length of up to 800 nm (Figure 10A,B). The high‐magnification images of EEG(TCPP) show graphene flakes with many wrinkles, and well‐defined dark lines that reveal few‐layer and bilayer graphene (Figure 10C,D).^[^ ^65^, ^123^, ^124^, ^125^ ^]^ EEG(Sn‐TCPP) flakes, on the other hand, appear smoother and exhibit no wrinkles and lengths smaller than EEG(TCPP) (Figure 11A,B). The edges of the EEG flakes dispersed in Sn‐TCPP are suggestive of monolayer graphene (Figure 11C,D).^[^ ^126^, ^127^ ^]^ The TEM results for EEG dispersed in DMF exhibit numerous flakes with sizes of ≈100 nm (Figure 12A). Similarly to EEG(TCPP), the EEG(DMF) reference sample also exhibits wrinkles (Figure 12A–C), as well as bilayer and few‐layer regions (Figure 12D). These results show that this method of production can yield both monolayer, bilayer, and few‐layer graphene.^[^ ^128^ ^]^

### 2.3 EEG Electronic Interactions with TCPP and Sn‐TCPP

After establishing that TCPP and Sn‐TCPP successfully produce highly stable EEG dispersions in water, we sought to investigate the nature of the interactions occurring between these two porphyrin molecules and the dispersed EEG flakes. For this purpose, fluorescence emission spectroscopy measurements were conducted for samples of EEG(TCPP)(2.5 mg ml^−1^) and EEG(Sn‐TCPP)(2.5 mg ml^−1^), to study the electronic communication between EEG graphene and porphyrin molecules in the excited state.^[^ ^129^, ^130^, ^131^ ^]^ The resulting fluorescence emission spectra are presented in **Figure** **13**. The porphyrin‐based emission of TCPP (Q (0–0): ≈650 nm and Q (0–1): ≈710 nm) was significantly quenched in the EEG(TCPP) spectrum.^[^ ^98^ ^]^ This quenching phenomenon indicates a strong interaction between the dispersed EEG flakes and the TCPP molecules.^[^ ^132^, ^133^ ^]^ It should be pointed out that, due to interference from the absorption of EEG at the excitation wavelength and the red shift of the Soret band, it is rather difficult to quantify the quenching of the porphyrin emission in the graphene‐based hybrid system.^[^ ^134^ ^]^ Notably, the observed porphyrin‐based emission quenching in the case of EEG(Sn‐TCPP) was lower, indicating weaker interactions between EEG and Sn‐TCPP. These results, which are consistent with the previously presented UV–vis absorption spectra (Figure 9), point to TCPP strongly interacting with the EEG particles, successfully dispersing them in water and formulating stable aqueous graphene dispersions.

To further support the electronic communication between the dispersed EEG flakes and the porphyrin molecules, the photoinduced dynamics of the excited state of EEG(TCPP) and EEG(Sn‐TCPP) samples were examined by time‐resolved spectroscopy. The fluorescence lifetime profiles of the photoexcited porphyrins and EEG‐porphyrin samples are presented in Figure S14 (Supporting Information) and were curve‐fitted using mono‐exponential decay kinetics. TCPP exhibited a lifetime (τ) of 9.3 ns, which is characteristic of a free base porphyrin, whereas EEG(TCPP) showed a reduced porphyrin lifetime (τ = 6.2 ns). It is therefore reasonable to assume a charge‐separation and/or energy transfer scenario in the EEG(TCPP) via the singlet excited state of TCPP. Notably, in the cases of EEG(Sn‐TCPP) and Sn‐TCPP both samples presented τ = 1.9 ns, further supporting the weaker interactions between EEG and Sn‐TCPP molecules in the excited state.

### 2.4 Characterization of Spray‐Coated EEG Films

The thickness values of the spray‐coated EEG(TCPP) and EEG(Sn‐TCPP) films were measured using a profilometer and are featured in **Table** **3**. Also, in the same table are listed the conductivity (σ) values, as measured via Van der Pauw Hall Effect measurements, and the Work Function (WF) values as measured by Ambient Photoemission Spectroscopy (APS).

**Table 3:** Measured thickness, conductivity (σ), and Work Function (WF) values of spray‐coated EEG(TCPP) and EEG(Sn‐TCPP) solid films.

Sample	Sprayed Volume [ml]	Thickness [nm]	Conductivity, σ [S/cm]	Work Function, WF [eV]
EEG(TCPP)	5 ml	105	0.22	4.79
	10 ml	180	14.95	4.81
EEG(Sn‐TCPP)	5 ml	75	0.09	4.47
	10 ml	110	2.82	4.54

As expected, the resulting film thickness increases with the volume of the deposited EEG dispersions. Larger thicknesses are achieved with EEG(TCPP), as it has a higher concentration than EEG(Sn‐TCPP), which is also evidenced by the higher UV–vis absorbance intensity (Figure 4). The conductivity of the films increases with the film thickness, specifically from 0.22 to 14.95 S/cm for EEG(TCPP) and 0.09 to 2.82 S/cm for EEG(Sn‐TCPP). Increasing the amount of the spray‐coated material increases the resulting film thickness and produces more compact and continuous layers, which, in turn, enhances the interaction between the deposited graphene flakes. This leads to a more efficient network of conductive pathways, which improves the film's electrical properties by lowering resistance and increasing overall conductivity. Lastly, thicker graphene films have fewer surface flaws and irregularities, resulting in a greater fraction of defect‐free regions. This structural integrity improves the film's conductive qualities.^[^ ^135^, ^136^ ^]^

The observed decreased WF_(ave)_ of 4.5 eV for EEG(Sn‐TCPP) compared to that of WF_(ave)_ of 4.8 eV for EEG(TCPP) can be attributed to the electronic and structural changes induced by the incorporation of Sn into the porphyrin framework.^[^ ^137^, ^138^ ^]^ The introduction of Sn, which forms coordination bonds with the nitrogen (N) atoms in the porphyrin ring, significantly alters the electronic distribution within the molecule. The Sn─N bonds are likely to change the electron density and shift the energy levels of the molecular orbitals. Tin's high atomic radius and low electronegativity can lead to a redistribution of electrons, impacting the π‐electron cloud of the conjugated porphyrin system. This reconfiguration affects the density of states near the Fermi level, thereby lowering the energy required to remove an electron. Consequently, the EEG(Sn‐TCPP) films exhibit lower WF values.

The recorded UV–vis absorption spectra of the spray‐coated EEG films are featured in Figure S11 (Supporting Information). For a deposited volume of 5 ml, both the EEG(TCPP) and the EEG(Sn‐TCPP) films exhibited a very similar absorption intensity. For a deposited volume of 10 ml, the EEG absorbance peak of the EEG(TCPP) film is stronger than that of EEG(Sn‐TCPP), which agrees with the stronger absorbance observed within the absorption spectrum of the EEG(TCPP) (2.5 mg ml^−1^) (Figures 2A,4A). For the EEG(TCPP) samples, a low‐intensity, broad shoulder is visible at ≈447 nm, attributed to the red‐shifted Soret band of TCPP.^[^ ^112^, ^113^, ^114^ ^]^ The EEG(Sn‐TCPP) films exhibit a low‐intensity signal at ≈420 nm owing to the presence of Sn‐TCPP which are not red‐shifted. The absorption spectra of the spray‐coated films are in accordance with the absorption spectra of the respective dispersions.

Finally, when building a solution‐processed device by sequentially depositing thin layers of functional materials on top of each other, knowing the surface roughness of each layer is important, as it affects the deposition of the next functional material and the interface between the two layers.^[^ ^139^, ^140^, ^141^, ^142^ ^]^ We therefore employed Atomic Force Microscopy (AFM) to investigate the resulting surface roughness of EEG(TCPP) and EG(Sn‐TCPP) when they are deposited with the same spray‐coating parameters. The obtained surface topography images along with the Root Mean Square (RMS) surface roughness values are included in Figures S12 and S13 (Supporting Information). EEG(TCPP) created films with smaller roughness values, with an average RMS of 30.6 nm for both sprayed volumes. EEG(Sn‐TCPP), on the other hand, yielded films with an average RMS of 90.5 nm. The smoother film formation in the case of EEG(TCPP) is attributed to the superior dispersibility and stability that TCPP achieves. The stronger π‐π interactions between EEG and TCPP, as evidenced by the fluorescence emission spectra (Figure 10), result in a higher‐concentration, more homogenous, and stable EEG dispersion, which in turn leads to smoother film formation when it is spray‐coated on the glass substrate. The lower RMS values of the EEG(TCPP) samples possibly contribute to their higher conductivities, as lower surface roughness directly affects charge carrier mobility by minimizing charge scattering and improving conductive pathways.^[^ ^143^ ^]^ This is not to say that the higher RMS values measured for EEG(Sn‐TCPP) would not be beneficial, since higher surface roughness is important for sensing applications and certain catalytic processes that require a larger active surface area.^[^ ^144^, ^145^ ^]^

## 3 Conclusion

In this work, we present a promising technique for preparing high‐concentration, stable graphene dispersions in water, by employing porphyrin‐based compounds as stabilizing agents. We successfully prepared dispersions of electrochemically exfoliated graphene (EEG) in aqueous solutions of TCPP and Sn‐TCPP. These aqueous dispersions remain stable for up to 1 week, as evidenced by the stability tests conducted using UV–vis absorption spectroscopy. Steady‐state and time‐resolved fluorescence emission studies revealed that TCCP interacts more strongly with the suspended EEG flakes, as evidenced by the higher emission quenching, thus leading to higher concentration and more stable EEG dispersions. The mechanism by which these two porphyrins keep the EEG flakes suspended in water can best be described by two interactions. First, EEG benzene rings interact via non‐covalent π‐π stacking with the aromatic rings of the porphyrin‐based compounds. Second, the hydrophilic carboxyl groups that TCPP and Sn‐TCPP possess, interact with the surrounding water molecules, thus keeping the EEG flakes suspended in the aqueous environment. We then expanded our investigation by preparing solid thin films of the prepared EEG aqueous dispersions via spray coating and exploring their optoelectronic properties and surface morphology. We observed that the EEG(TCPP) produced films with lower surface roughness values and higher conductivity compared to EEG(Sn‐TCPP). The lower RMS values of the EEG(TCPP) films contribute to higher conductivities, as lower surface roughness minimises charge scattering and improves conductive pathways. The EEG(Sn‐TCPP) films have an average WF value that is ≈0.3 eV lower than that of EEG(TCPP), which we attribute to the presence of the Sn metallic centre.

## 4 Experimental Section

### Materials and Equipment

To produce electrochemically exfoliated graphene (EEG), graphite foil 99.8%, platinum (Pt) foil 99.9% and ammonium sulfate, (NH_4_)_2_SO_4_ ≥99.0% were purchased from Sigma Aldrich. The produced EEG powder was characterised using ATR‐IR and Raman spectroscopy to evaluate its quality. The ultrapure water used for the preparation of aqueous graphene dispersions was purchased from Honeywell. The synthesis of the porphyrin and metalloporphyrin compounds [H_2_TCPP]Na_4_, [Sn(ΟH)_2_TCPP]Na_4_, [H_2_TMPyP]I_4_, [Sn(Cl_2_)TMPyP]Cl_4_ and Sn(OH)_2_TPyP were synthesised according to known experimental procedures.^[^ ^98^, ^99^, ^100^, ^101^ ^]^ Their successful preparation was verified based on their recorded ^1^H‐NMR. The ^1^H‐NMR spectrum of [Sn(ΟH)_2_TCPP]Na_4_ was unable to be obtained due to solubility issues, therefore successful preparation was confirmed via MALDI‐TOF mass spectrometry. NMR spectra were recorded on Bruker AMX‐500 MHz and Bruker DPX‐300 MHz spectrometers. All measurements were carried out at room temperature in a deuterated solvent using residual protons as an internal reference. Mass spectra were recorded on a Bruker ultrafleXtreme MALDI‐TOF/TOF spectrometer. N,N’‐Dimethylformamide (DMF) 99.8% for the preparation of the reference samples was purchased from Sigma Aldrich. The electrochemical exfoliation of graphite foil was performed using a regulated DC power supply by UNIT‐T and an Elma S 30H ultrasonic cleaning bath (80 W, 37 kHz). The produced Electrochemically Exfoliated Graphene (EEG) was dispersed in aqueous solutions of the five porphyrins and in DMF as a reference sample using a Hielscher UP200Ht ultrasonic probe (200 W, 26 kHz, 100% Amplitude). Subsequent centrifugations were carried out in a Hettich UNIVERSAL 320 centrifuge. ATR‐IR transmittance spectra were obtained with a Bruker Vertex 70v FT‐IR vacuum spectrometer equipped with an A225/Q Platinum ATR unit with single reflection diamond crystal, which allows the infrared analysis of unevenly shaped solid samples and liquids through total reflection measurements, in a spectral range of 7500–380 cm^−1^. Raman spectra were obtained at room temperature using a modified LabRAM HR Raman Spectrometer (HORIBA Scientific, Kyoto, Japan). Raman excitation was achieved with a 532 nm central wavelength solid‐state laser module with a maximum laser output power of 90 mW. The microscope was coupled with a 50x microscopic objective lens with 0.5 numerical aperture and 10.6 mm working distance (LMPlanFLN 50X/0.5, Olympus, Tokyo, Japan) that delivers the excitation light and collects the Raman signals. The laser spot size was ≈1.7 µm laterally and ≈2 µm axially. A 600 grooves/mm grating was used, resulting in a Raman spectral resolution of ≈2 cm^−1^. UV–vis absorption spectra were obtained with a Shimadzu UV‐2401 PC Recording Spectrophotometer with a maximum range of 190–1000 nm. XRD patterns were recorded using a RIGAKU (Tokyo, Japan) D/MAX‐2500 powder diffractometer equipped with a monochromated Cu K_α_ radiation (λ = 1.5418 Å). The emission spectra were measured on a JASCO FP‐6500 fluorescence spectrophotometer equipped with a red‐sensitive WRE‐343 photomultiplier tube (wavelength range: 200–850 nm). For the emission measurements, iso‐absorbing solutions were prepared with respect to the Soret porphyrin absorption peak in water. For the iso‐absorbing solution preparation, the EEG‐attributed absorption was subtracted, providing the porphyrin Soret band‐attributed absorption. Afterwards, the Soret band was excited for each solution (i.e., 414 nm for the TCPP, 447 nm for the EEG(TCPP), 420 nm for the Sn‐TCPP, and 420 nm for EGG(Sn‐TCPP). SEM images were obtained with a JEOL (JSM‐IT700HR‐LV) Scanning Electron Microscope with an acceleration voltage of 15.0 kV. TEM imaging was performed using a JEOL (Tokyo, Japan) JEM2100 200 kV analytical electron microscope, equipped with a LaB_6_ electron gun. EEG solid film thickness measurements were performed with a Bruker DektakXT profilometer. AFM was performed on the EEG films with an XE‐7 Parks System atomic microscope, using a non‐contact mode, to obtain surface topography images and surface roughness expressed through Root Mean Square (RMS) values. The scanned surface area was 20 × 20 µm. Dark Work Function (WF) measurements were performed with an APS04 N2‐RH system (KP Technology). More specifically, the contact potential difference (CPD) was measured using a vibrating Kelvin probe gold alloy (2 mm in diameter). The absolute WF of the tip was estimated to be ≈4.54 ± 0.06 eV, which was calibrated by measuring a silver reference and calculating its absolute WF by APS.

### Preparation of Electrochemically Exfoliated Graphene (EEG)

The graphene used in this work was produced in‐lab via cathodic electrochemical exfoliation of graphite foil. This setup uses graphite foil as the cathode and platinum foil as the anode. The electrodes were submerged in an aqueous (NH_4_)_2_SO_4_ solution (0.1 m) which was placed in an ultrasonic cleaner. A voltage of 10 V was applied across the electrodes for 10 min. The application of ultrasonic frequencies during this process enhances the efficiency of exfoliation, by aiding in the detachment of the produced graphene flakes from the bulk graphite foil. The produced electrochemically exfoliated graphene (EEG) was washed thoroughly with ultrapure water and dried in an oven (50 °C) overnight. Electrochemical exfoliation with these parameters, i.e., voltage and electrolyte solution, typically has a yield of over 75%.^[^ ^48^ ^]^ The EEG powder was characterised using ATR‐IR and Raman spectroscopy and XRD to evaluate its quality, and chemical makeup and establish that it was not oxidized during the electrochemical exfoliation process (Figure S1, Supporting Information).

### Preparation & Characterization of EEG Dispersions in Aqueous Porphyrin Solutions

**Figure 6:**
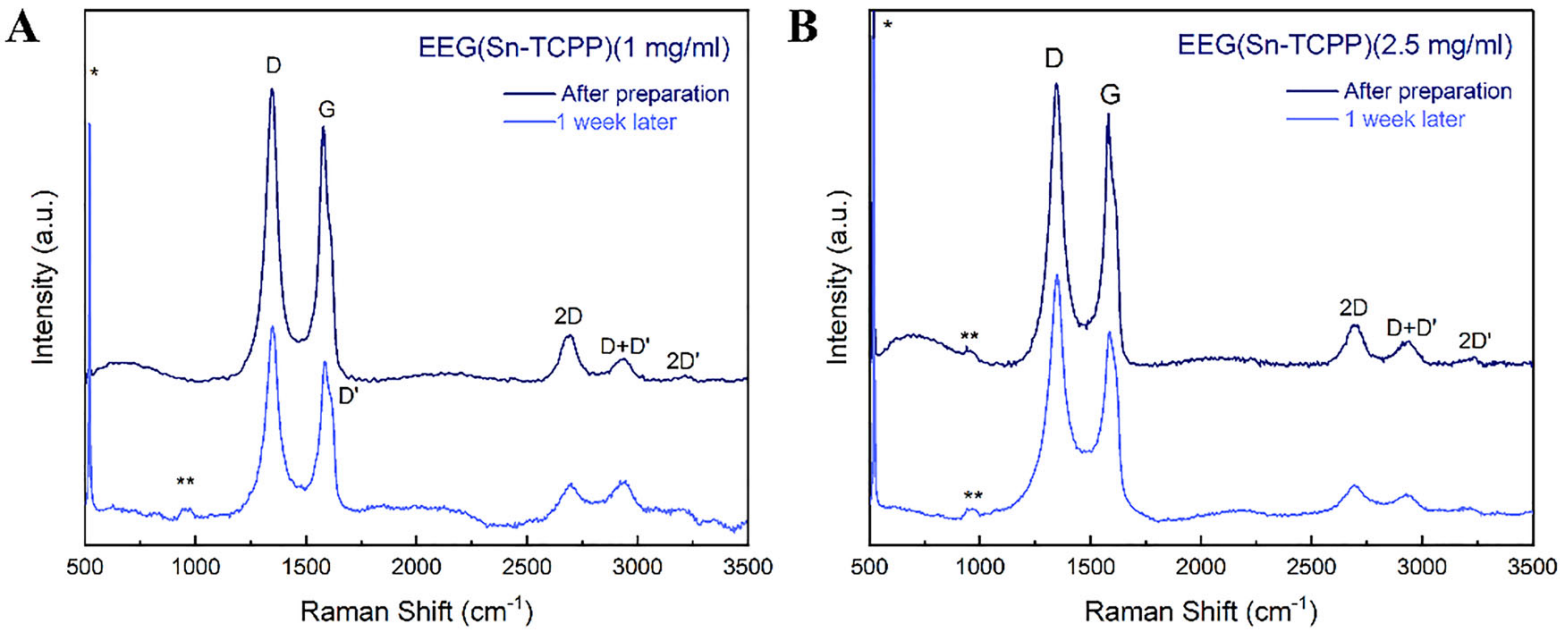
Raman spectra of A) EEG(Sn‐TCCP) (1 mg ml^−1^) and B) EEG(Sn‐TCPP) (2.5 mg ml^−1^) immediately after dispersion and 1 week after preparation.

The water‐dispersibility of the produced EEG was tested in aqueous solutions of five porphyrin‐based compounds: [H_2_TCPP]Na_4_, [Sn(OH)_2_TCPP]Na_4_, [H_2_TMPyP]I_4_, [SnCl_2_TMPyP]Cl_4_ and Sn(OH)_2_TMPyP (Figure 1). To this end, EEG (1 mg ml^−1^) was dispersed in an aqueous solution of each porphyrin (8 ml, 10^−5^ m) using an ultrasonic tip (100% A, 26 KHz, Pmax = 200 W) for 1.5 h. Each dispersion was then centrifugated (2000 rpm, 492 g, 30 min) to remove any undispersed particles or aggregates (Figure S8, Supporting Information). The UV–vis absorption spectrum of each dispersion was recorded immediately after preparation, to determine the dispersibility of EEG in each porphyrin solution. For the samples that displayed successful dispersion of EEG, additional UV–vis absorption spectra were recorded again after 24 h, 48 h, and 1 week, to assess the stability of the suspended EEG flakes over time. Because graphene was readily dispersed in DMF, EEG (1 mg ml^−1^) was dispersed in DMF (8 ml) following the same ultrasonication and centrifugation parameters, to be used as a reference sample for the dispersibility and stability of EEG in the porphyrin solutions (Figure S8, Supporting Information). All EEG dispersion samples were diluted 50 times before measuring their UV–vis absorbance. The porphyrin solutions measured as reference samples were diluted 20 times to obtain a more prominent and identifiable characteristic maximum absorption peak. The UV–vis absorption spectra of EEG (1 mg ml^−1^) in porphyrin solutions and DMF are presented in Figures 5 and 6. After it was established that [H_2_TCPP]Na_4_, [Sn(OH)_2_TCPP]Na_4_ yielded high‐concentration and stable EEG dispersions, a second series of dispersion experiments was performed in which the concentration of EEG was increased from 1 to 2.5 mg ml^−1^. EEG (2.5 mg ml^−1^) dispersions were prepared in TCPP (10^−5^ m) and Sn‐TCPP (10^−5^ m) following the same parameters described previously. EEG(DMF) (2.5 mg ml^−1^) was prepared as a reference sample. Digital photographs (Figures S9 and S10, Supporting Information) and UV–vis absorption spectra (Figure 9 ) were obtained as before to assess the dispersibility and stability of 2.5 times the amount of EEG in the same porphyrin solutions.

**Figure 7:**
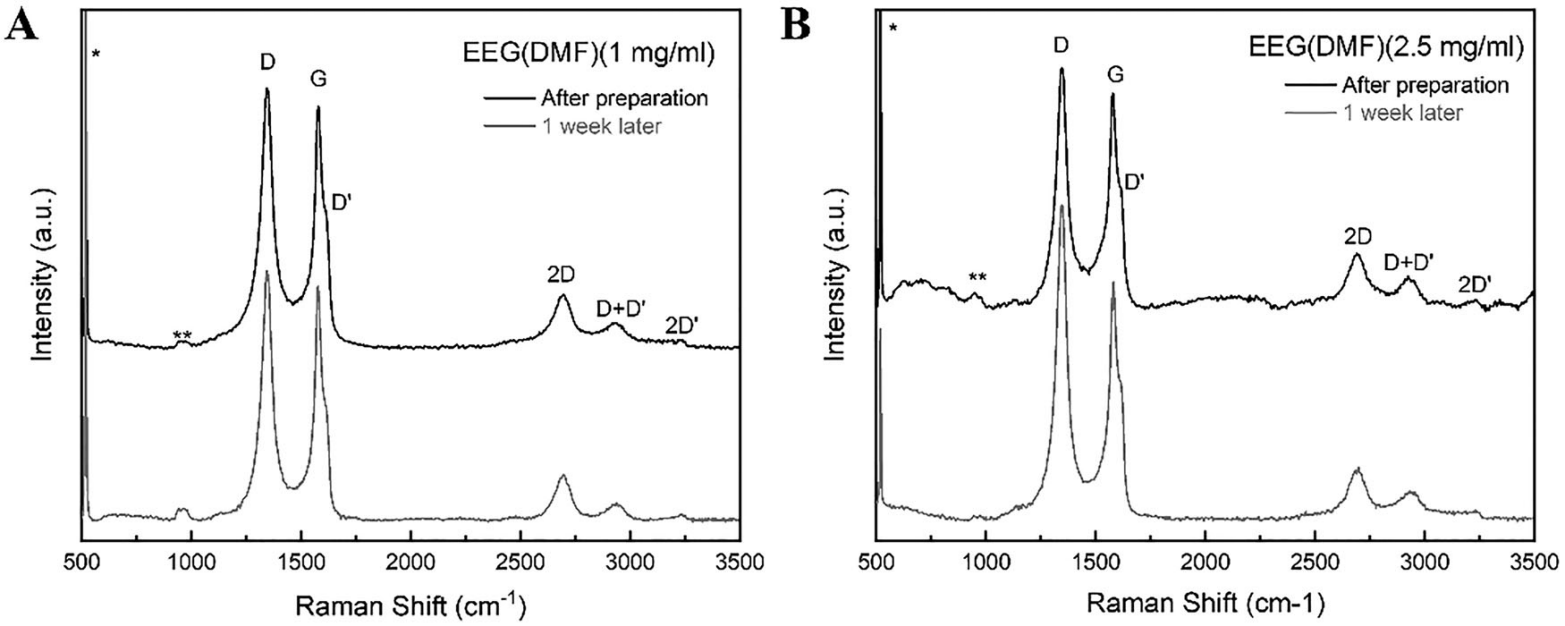
Raman spectra of A) EEG(DMF) (1 mg ml^−1^) and B) EEG(DMF) (2.5 mg ml^−1^) were used as a reference immediately after dispersion and 1 week after preparation.

**Figure 8:**
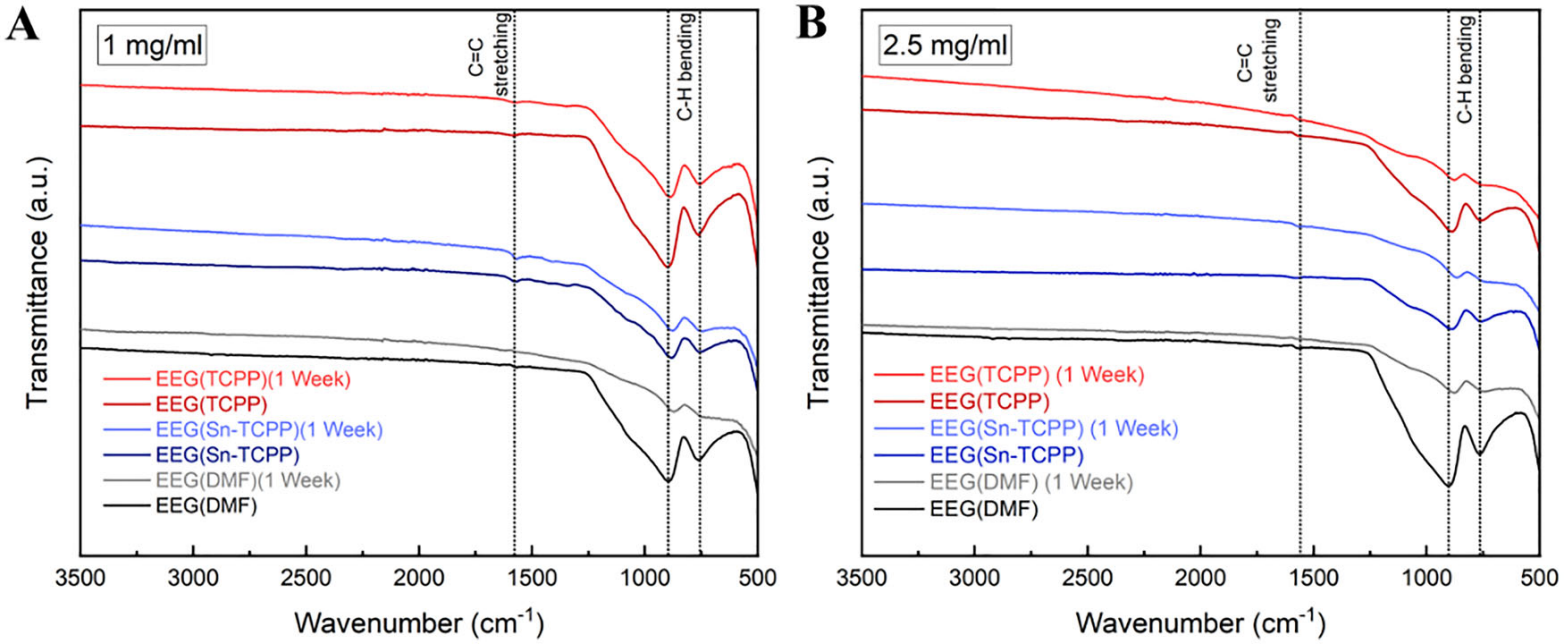
ATR‐IR spectra of A) EEG (1 mg ml^−1^) and B) EEG (2.5 mg ml^−1^) dispersed in TCCP, Sn‐TCPP, and DMF immediately after preparation and 1 week later.

**Figure 9:**
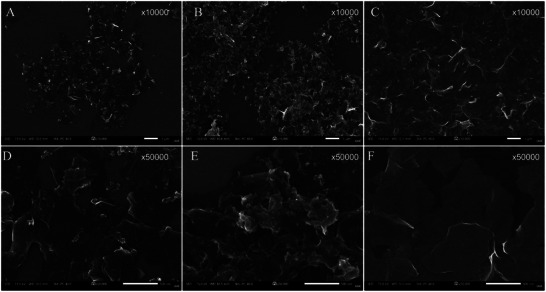
SEM images of EEG flakes dispersed in TCPP (A,D), Sn‐TCPP (B,E), and DMF (C,F).

**Figure 10:**
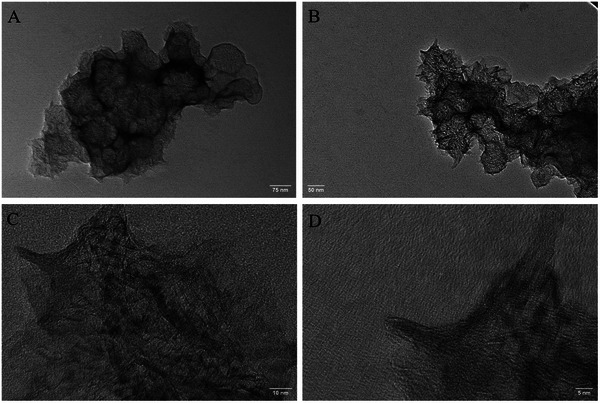
TEM images were obtained for EEG(TCPP). The scale bars are: A) 75 nm, B) 50 nm, C) 10 nm and D) 5 nm.

**Figure 11:**
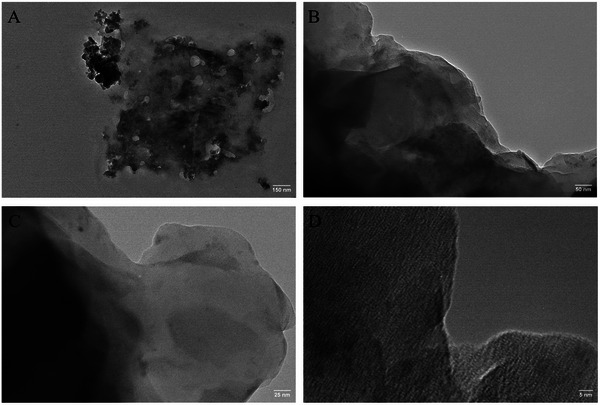
TEM images were obtained for EEG(Sn‐TCPP). The scale bars are: A) 150 nm, B) 50 nm, C) 25 nm and D) 5 nm.

**Figure 12:**
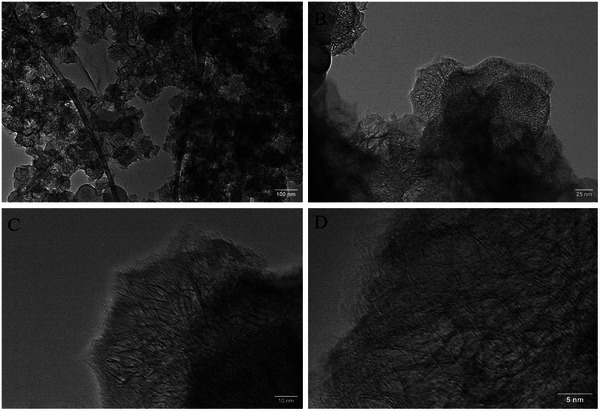
TEM images were obtained for EEG(DMF) reference. The scale bars are: A) 100 nm, B) 25 nm, C) 10 nm and D) 5 nm.

**Figure 13:**
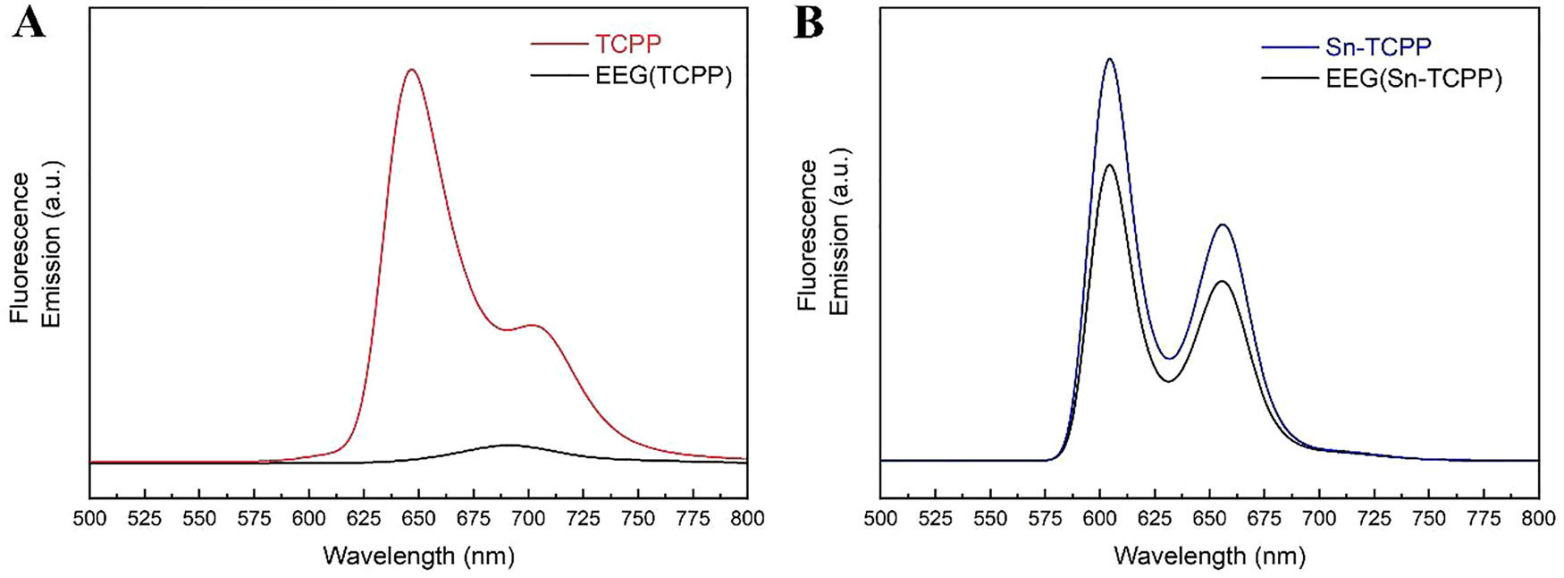
Fluorescence emission spectra of A) EEG(TCPP) with TCPP as a reference (both spectra had Absorbance = 0.1 at the Soret band) and B) EEG(Sn‐TCPP) with Sn‐TCPP as a reference (both spectra had Absorbance = 0.1 at the Soret peak). In all cases the samples were excited at the corresponding Soret band.

### Preparation of and Characterization of EEG Solid Thin Films

After achieving high‐concentration EEG dispersions in aqueous solutions of [H_2_TCPP]Na_4_ and [Sn(OH)_2_TCPP]Na_4_, solid thin films of graphene were prepared via spray‐coating on glass substrates and characterised in terms of their morphological, electrical and optoelectronic properties. First, glass substrates (7.5 × 2.5 cm) were cleaned following a four‐step cleaning method.^[^ ^146^ ^]^ They were then treated with O_2_‐plasma (50 W, 5 min) to increase the surface hydrophilicity and promote the formulation of a continuous solid film during the material deposition.^[^ ^147^ ^]^ The glass substrates were placed on a hotplate (70 °C) to promote solvent evaporation during the spray‐coating process. EEG(TCPP)(2.5 mg ml^−1^) and EEG(Sn‐TCPP)(2.5 mg ml^−1^) were then spray coated onto the prepared glass substrates (height: 15 cm, length: 8 cm, carrier gas: air, carrier gas pressure: 2 bar, spray coating rate: 0.1 ml s^−1^, sprayed area: ≈8 × 3 cm = 24 cm^2^). 5 ml (≈0.25 ml cm^−2^) and 10 ml (≈0.4 ml cm^−2^) of each EEG dispersion were deposited onto a glass substrate. The UV–vis absorption spectra of all sprayed EEG films were recorded (Figure S11, Supporting Information). The thickness (t) of each film was acquired through profilometer measurements. AFM was employed to determine the surface morphology and roughness of the prepared EEG films. Obtained surface topography images along with the Root Mean Square (RMS) surface roughness values are included in Figures S12 and S13 (Supporting Information). WF values were attained via APS, while σ values were recorded using a Van der Pauw Hall Effect measuring system.

## Conflict of Interest

The authors declare no conflict of interest.

## Author Contributions

K.A. and E.S. contributed equally to this work. K.A. and E.S. contributed to the research conceptualization and methodology, the execution of all experiments, the analysis of obtained data, the interpretation of the results, and the writing and visualization of the initial manuscript draft. K.A. and E.S. performed dispersibility and stability experiments and recorded and analysed UV–vis absorption spectra. K.A. contributed to the research project conceptualization and the experimental plan design and performed and analysed all Hall Effect measurements. E.S. prepared EEG via electrochemical exfoliation and performed all AFM measurements. N.T. performed all APS measurements and analysed the results. K.R. contributed to the review and editing of the draft. C.P. performed and analysed all profilometer measurements. C.P. and N.T. contributed to the execution of characterization experiments, the analysis of experiment results, and the visualization of the manuscript. A.K. synthesised [Sn(OH)_2_TCPP]Na_4,_ K. A. synthesised [H_2_TCPP]Na_4_. E.T. synthesised [H_2_TMPyP]I_4_ and [SnCl_2_TMPyP]Cl_4_. G.L. synthesised Sn(OH)_2_TPyP. E.N. performed and analysed all fluorescence experiments and contributed to the draft editing. G.C. contributed to result analysis, mechanism discussion methodology, and editing A.G.C. and E.K. provided resources and supervision. All authors revised and reviewed the manuscript.

## Supporting information



Supporting Information

## Data Availability

The data that support the findings of this study are available from the corresponding author upon reasonable request.
