# Supplementary material for: Enhancing the Dispersibility and Stability of Graphene in Water Using Porphyrin‐Based Compounds
Source: Small Methods. 2025 Mar 10;9(8):2401431. doi: 10.1002/smtd.202401431 (PMC12391633; doi:10.1002/smtd.202401431)
Supplement: Supplementary file 1 — Supporting Information [file SMTD-9-2401431-s001.docx]

Supporting Information

**Enhancing the Dispersibility and Stability of Graphene in Water Using Porphyrin-based Compounds^†^**

*Katerina Anagnostou, Evangelos Sotiropoulos, Nikolaos Tzoganakis, Christos Polyzoidis, Konstantinos Rogdakis, Anna Katsari, Katerina Achilleos, Evitina Triantafyllou, Georgios Landrou, Emmanouil Nikoloudakis, Georgios Charalambidis^*^, Athanassios G. Coutsolelos_,_ Emmanuel Kymakis^*^*

**
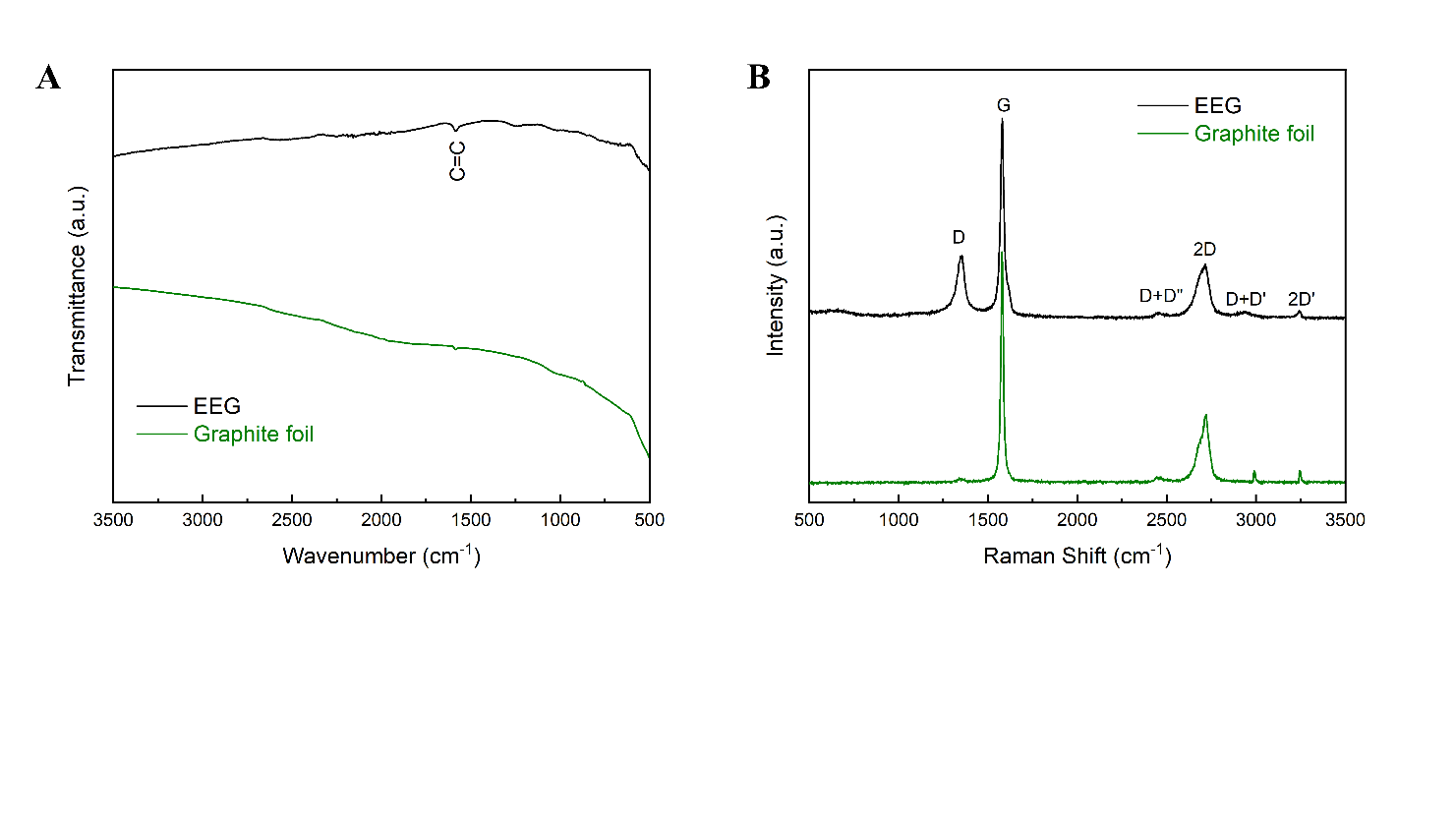
**

**Figure S1.** A) ATR-IR and B) Raman spectra of produced EEG powder and graphite foil precursor.


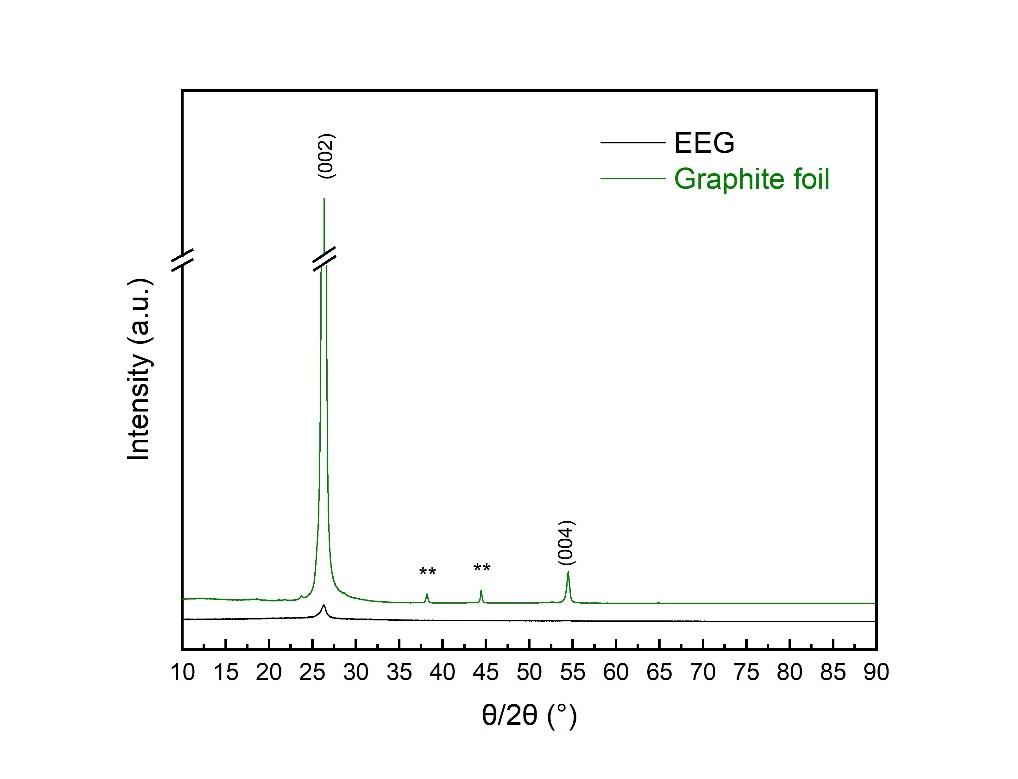


**Figure S2.** XRD patterns of EEG powder and graphite foil precursor.


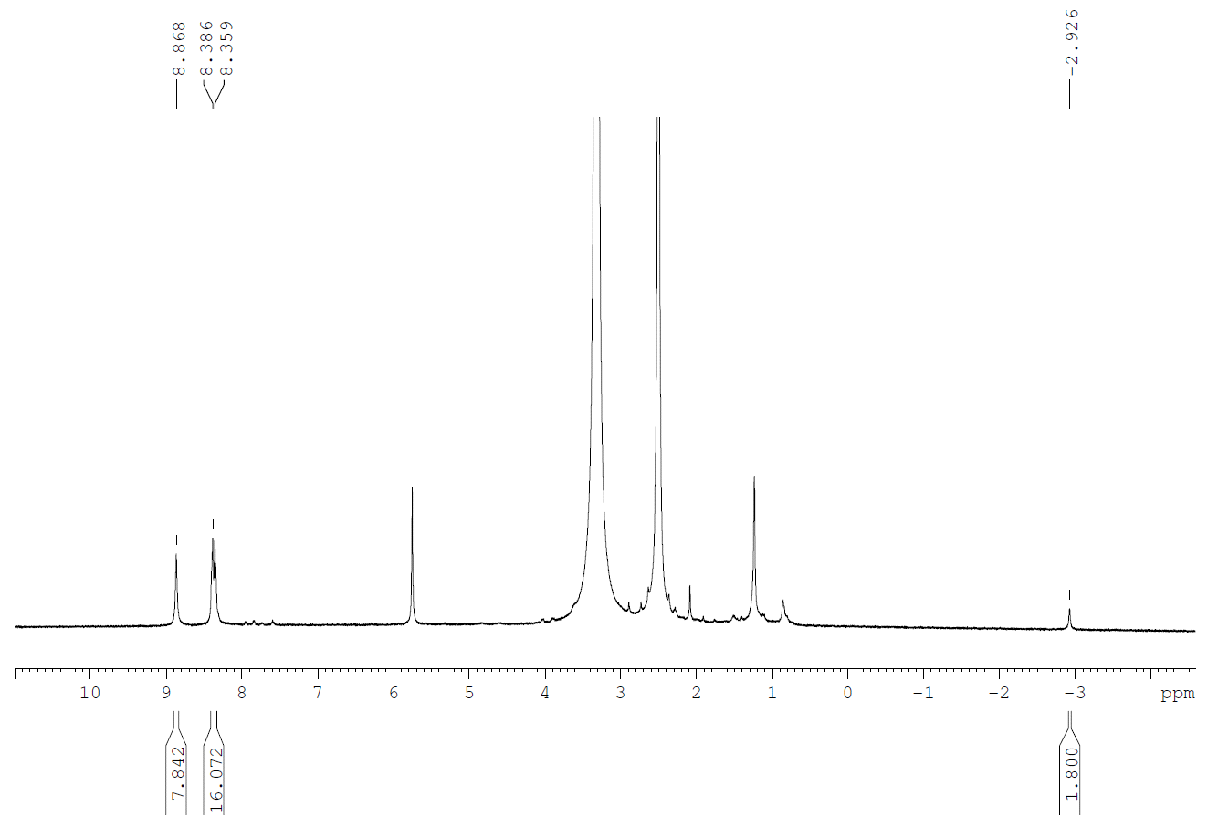


**Figure S3.** ^1^H NMR spectrum of compound [H_2_TCPP]Na_4_ or TCPP in d6-DMSO.


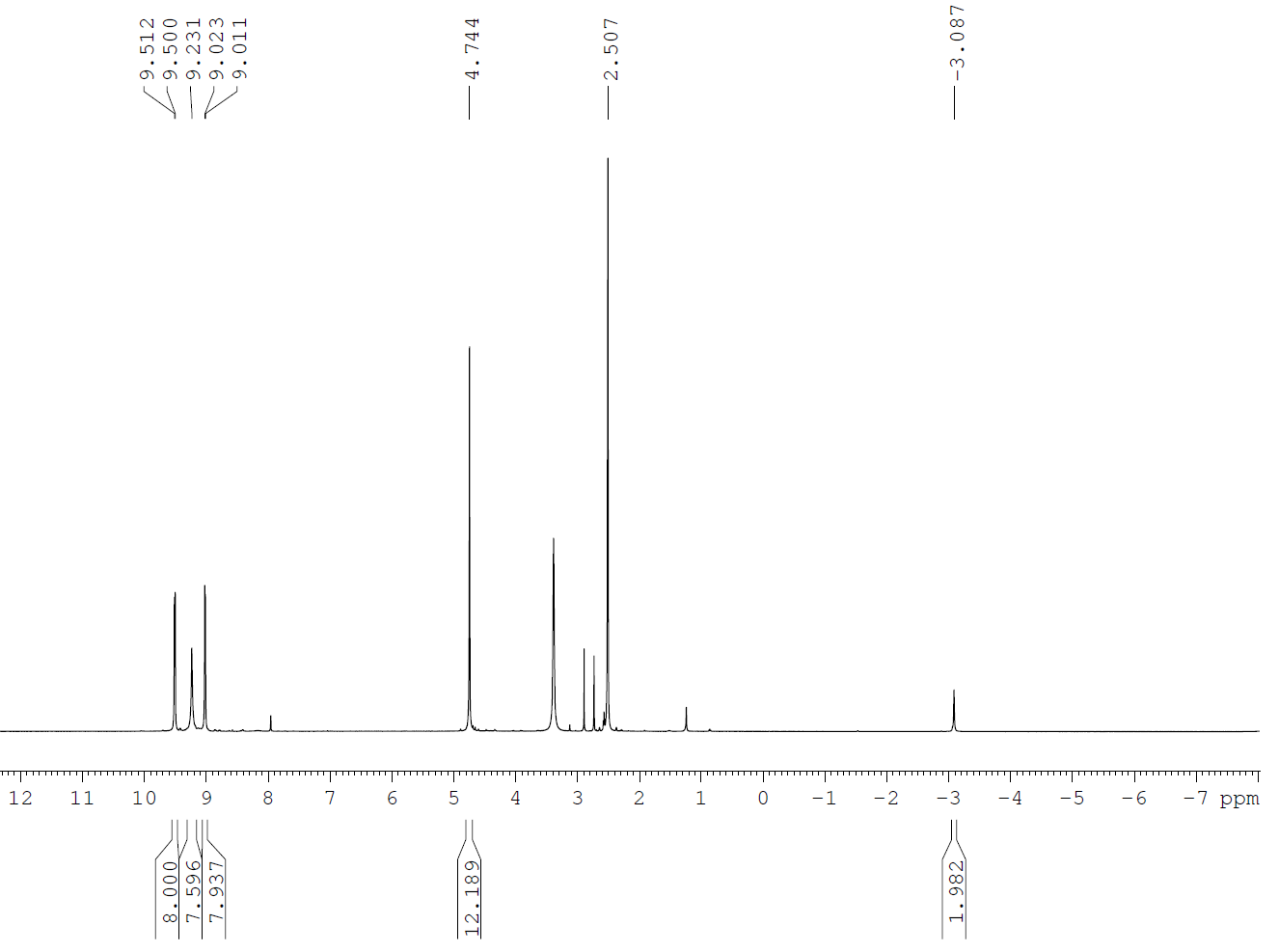


**Figure S4.** ^1^H NMR spectrum of compound [H_2_TMPyP]I_4_ in d6-DMSO.


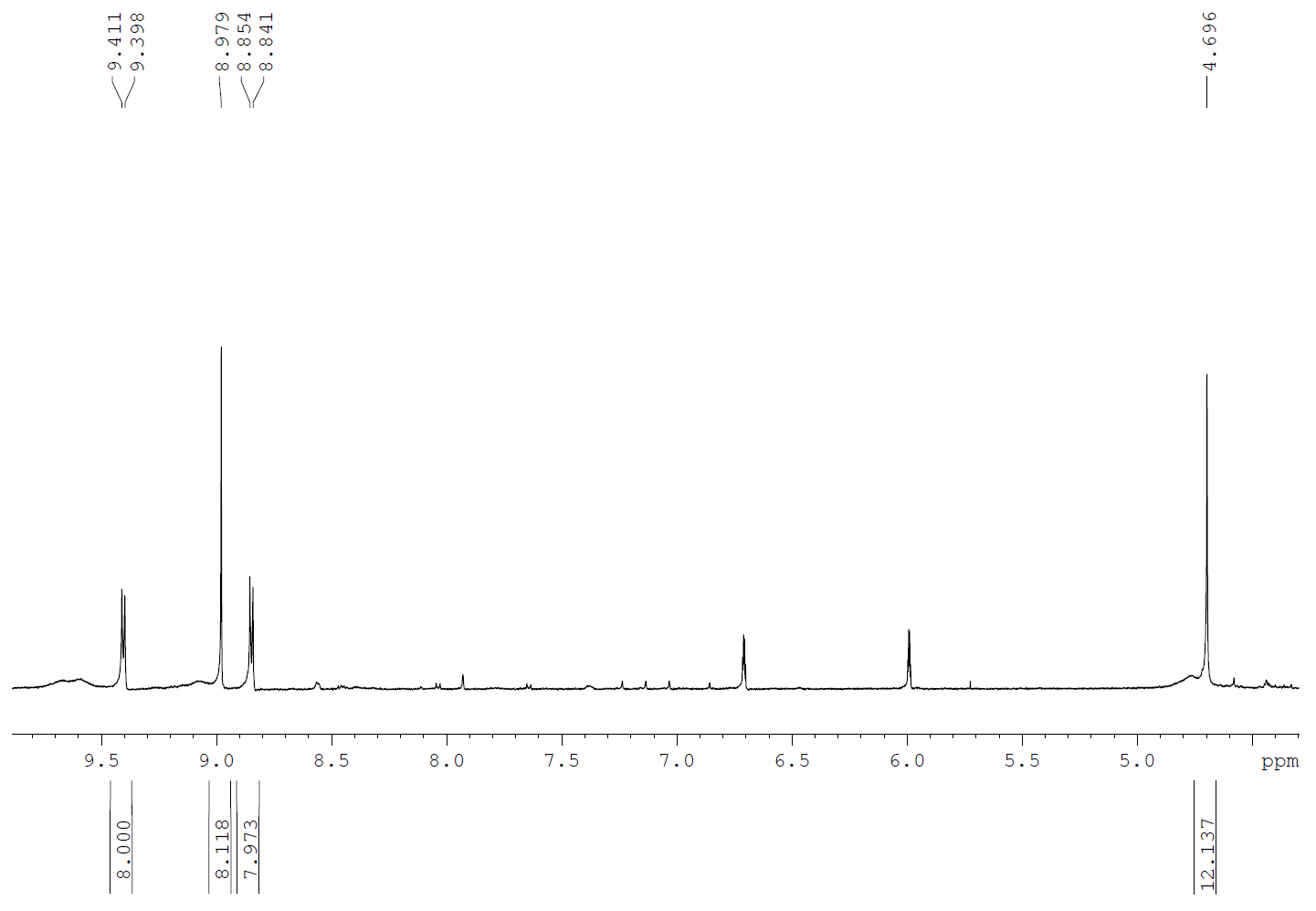


**Figure S5.** ^1^H NMR spectrum of compound [Sn(Cl_2_)TMPyP]Cl_4_ in d6-DMSO.


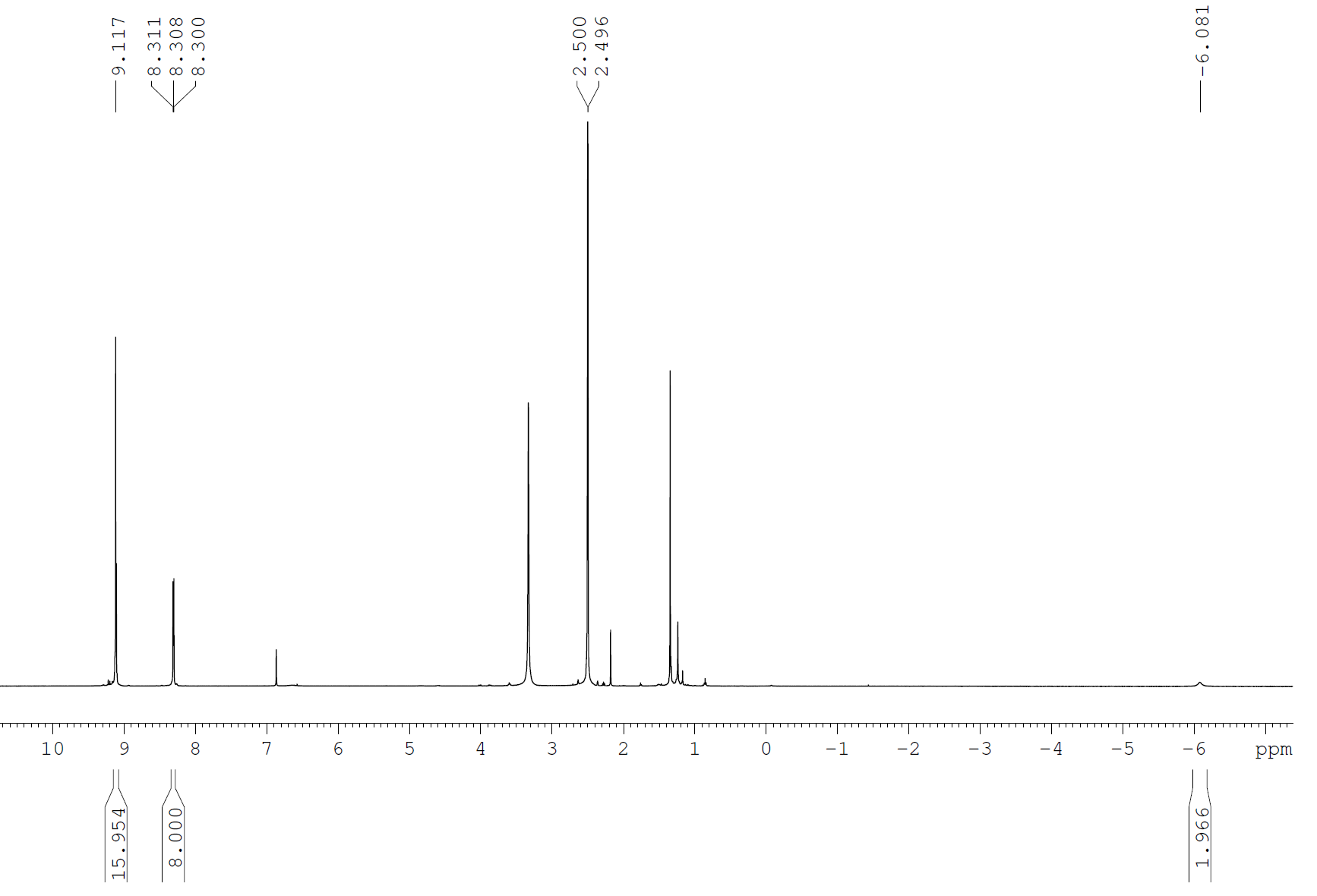


**Figure S6.** ^1^H NMR spectrum of compound Sn(OH)_2_TPyP in d6-DMSO.


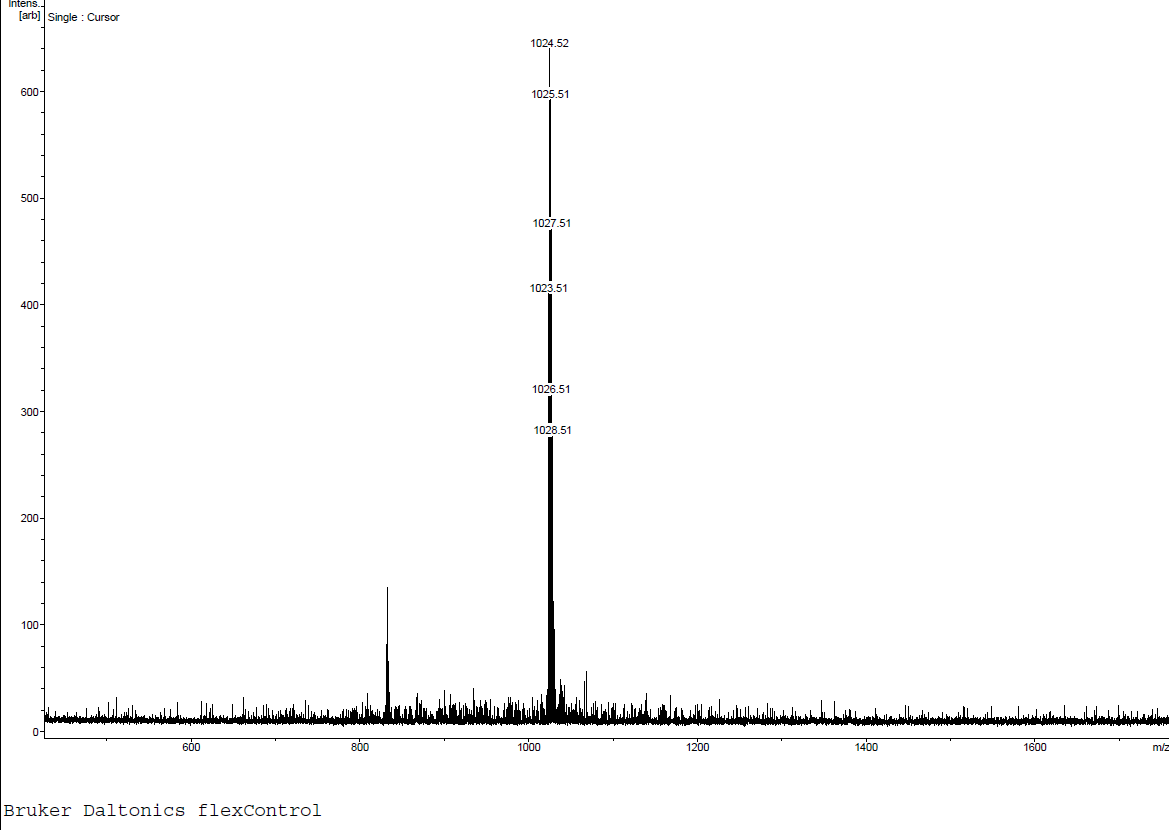


**Figure S7.** MALDI-TOF spectrum of compound [Sn(OH)_2_TCPP]Na_4_.


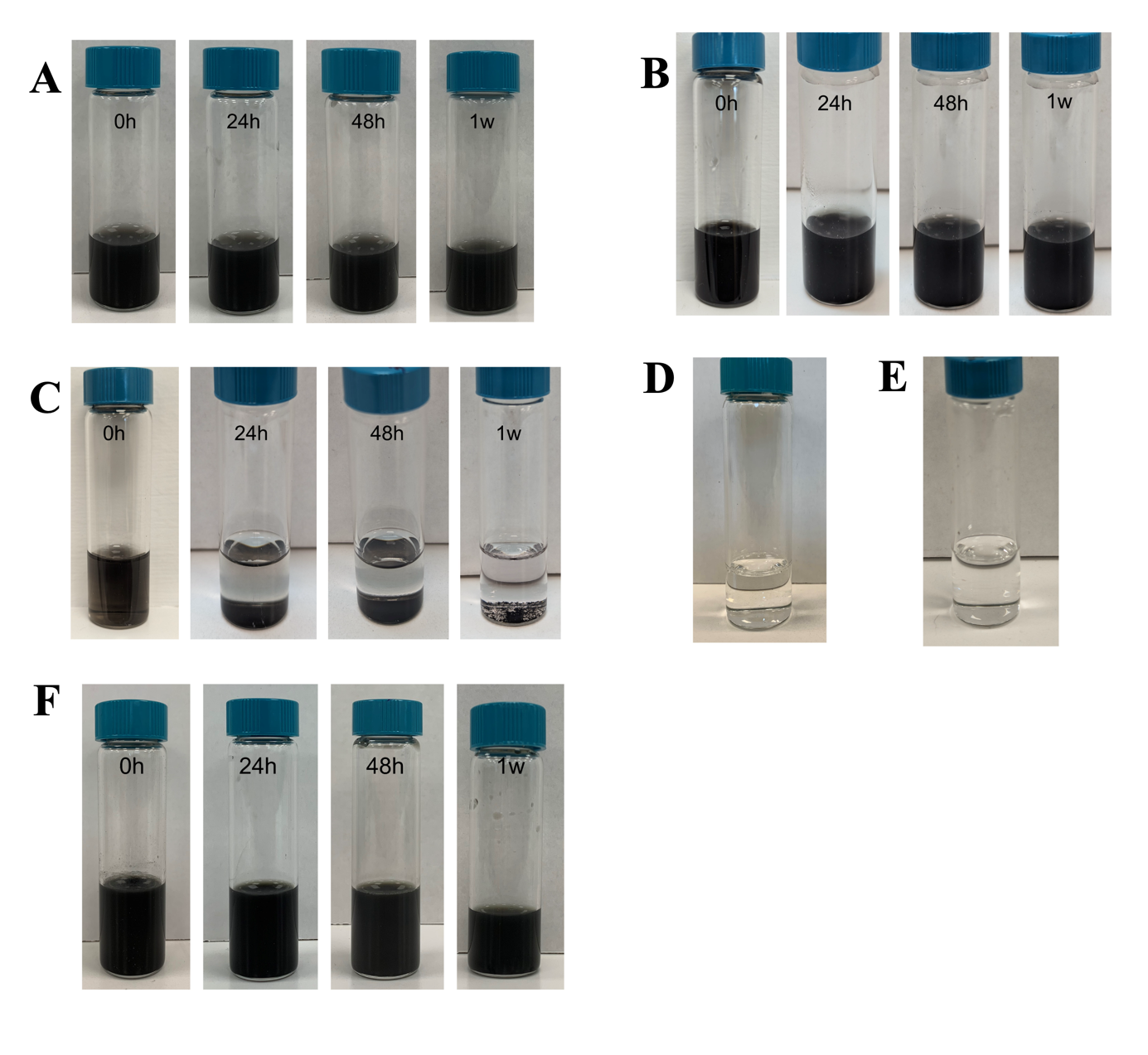


**Figure S8.** Digital photographs of EEG (1 mg/ml) dispersed in: A) [H_2_TCPP_4_]Na_4_ or (TCPP); B) [Sn(OH)_2_TCPP_4_]Na_4_ or (Sn-TCPP); C) [SnCl_2_TMPyP]Cl_4_, taken at 0h, 24h, 48h, and 1 week post-centrifugation. Both TCPP and Sn-TCPP showed successful dispersion and stability up to 1 week. C) [SnCl_2_TMPyP]Cl_4_ yielded poor dispersion and stability. Photographs of supernatants from EEG (1 mg/ml) dispersed in D) [TMPyP]I_4_ and E) Sn(OH)_2_TMPyP confirm poor dispersibility in these solutions. F) Photographs of EEG (1 mg/ml) dispersed in DMF, taken at 0h, 24h, 48h, and 1 week, were used as reference due to DMF’s stable dispersion properties for graphene.


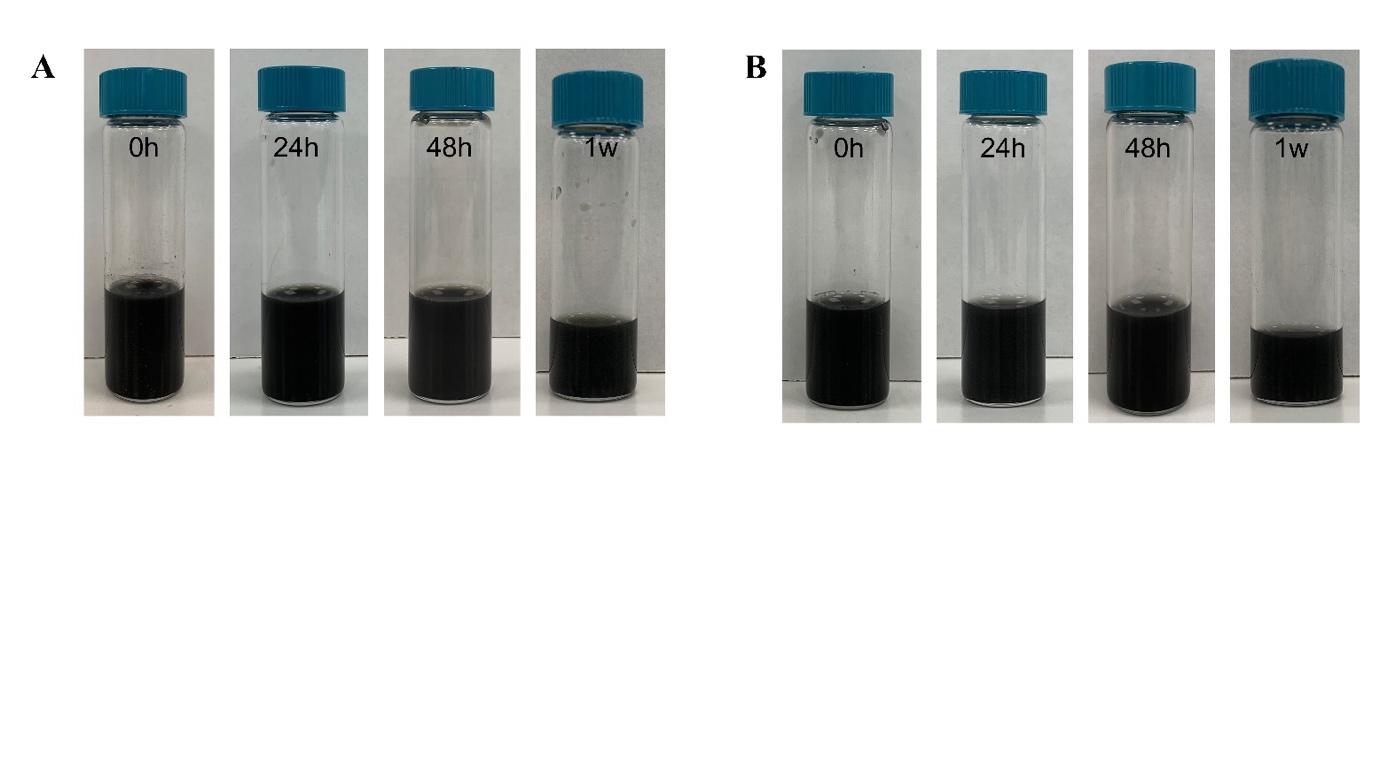


**Figure S9.** Digital photographs of EEG (2.5 mg/ml) dispersed in: A) TCPP and B) Sn-TCPP taken immediately after preparation (0h), after 24h, after 48h and after 1 week. As with EEG (1 mg/ml), both porphyrin aqueous solutions successfully dispersed EEG and exhibited high dispersion stability for up to a week.

**
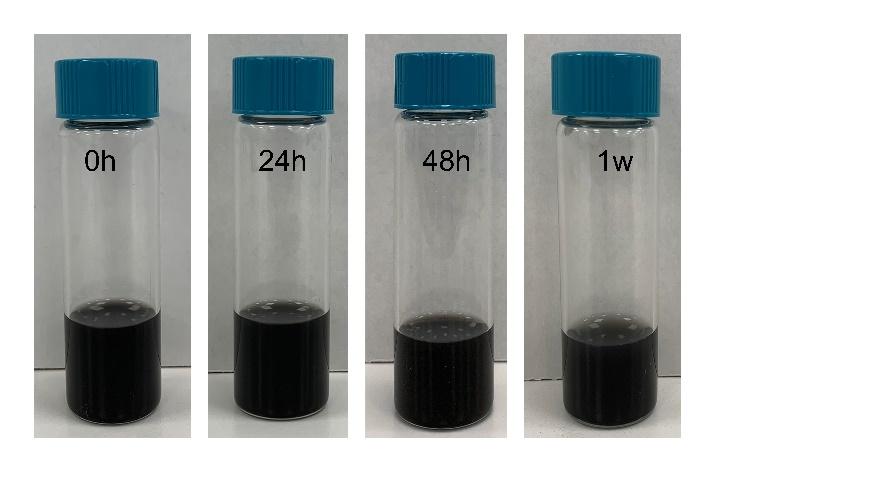
**

**Figure S10.** Digital photographs of the EEG(DMF) (2.5 mg/ml) reference sample taken immediately after preparation (0h), 24h, 48h and 1 week.


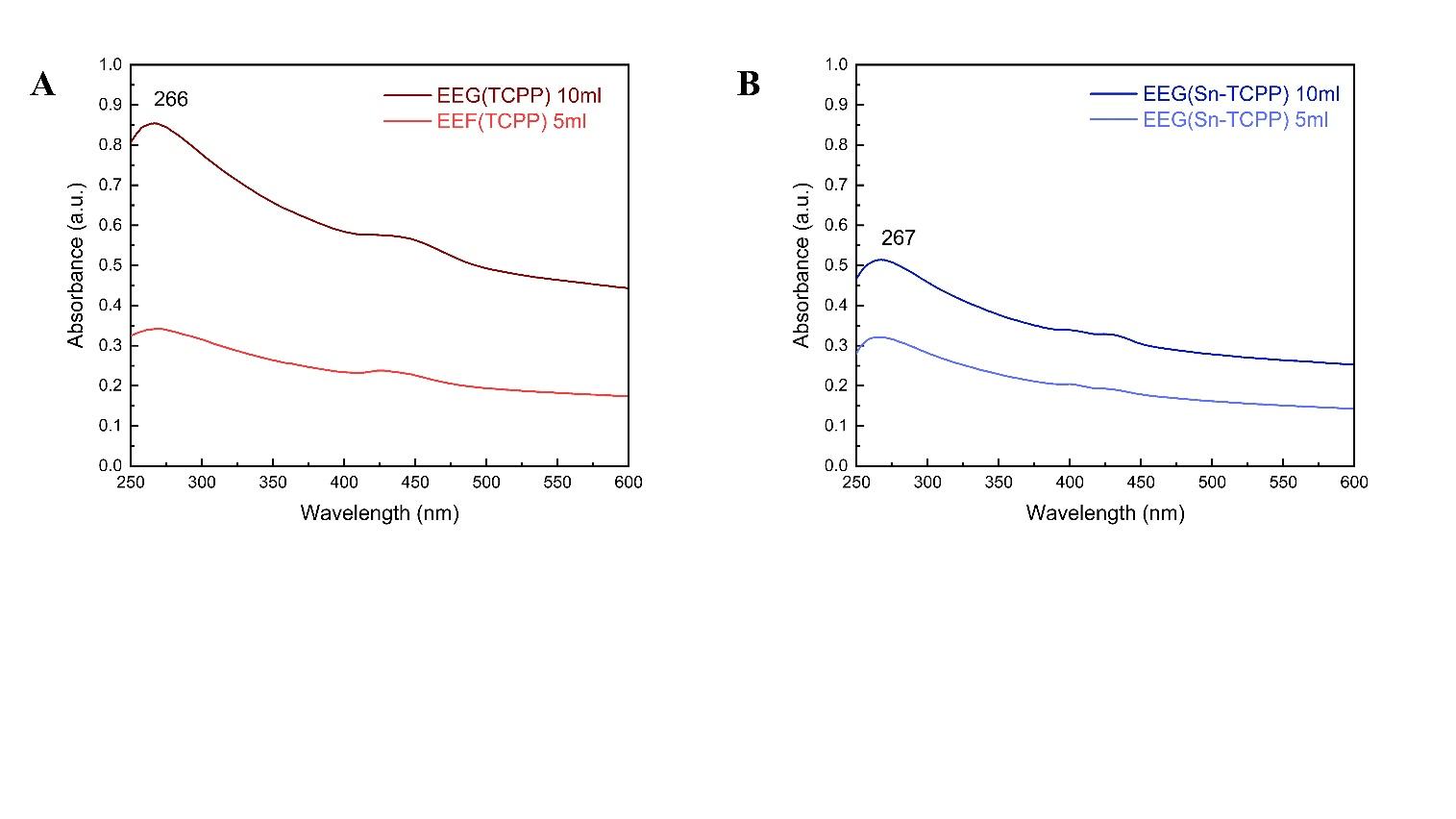


**Figure S11**. UV-Vis absorption spectra of A) EEG(TCPP) and B) EEG(Sn-TCPP) films prepared by spray-coating.

**
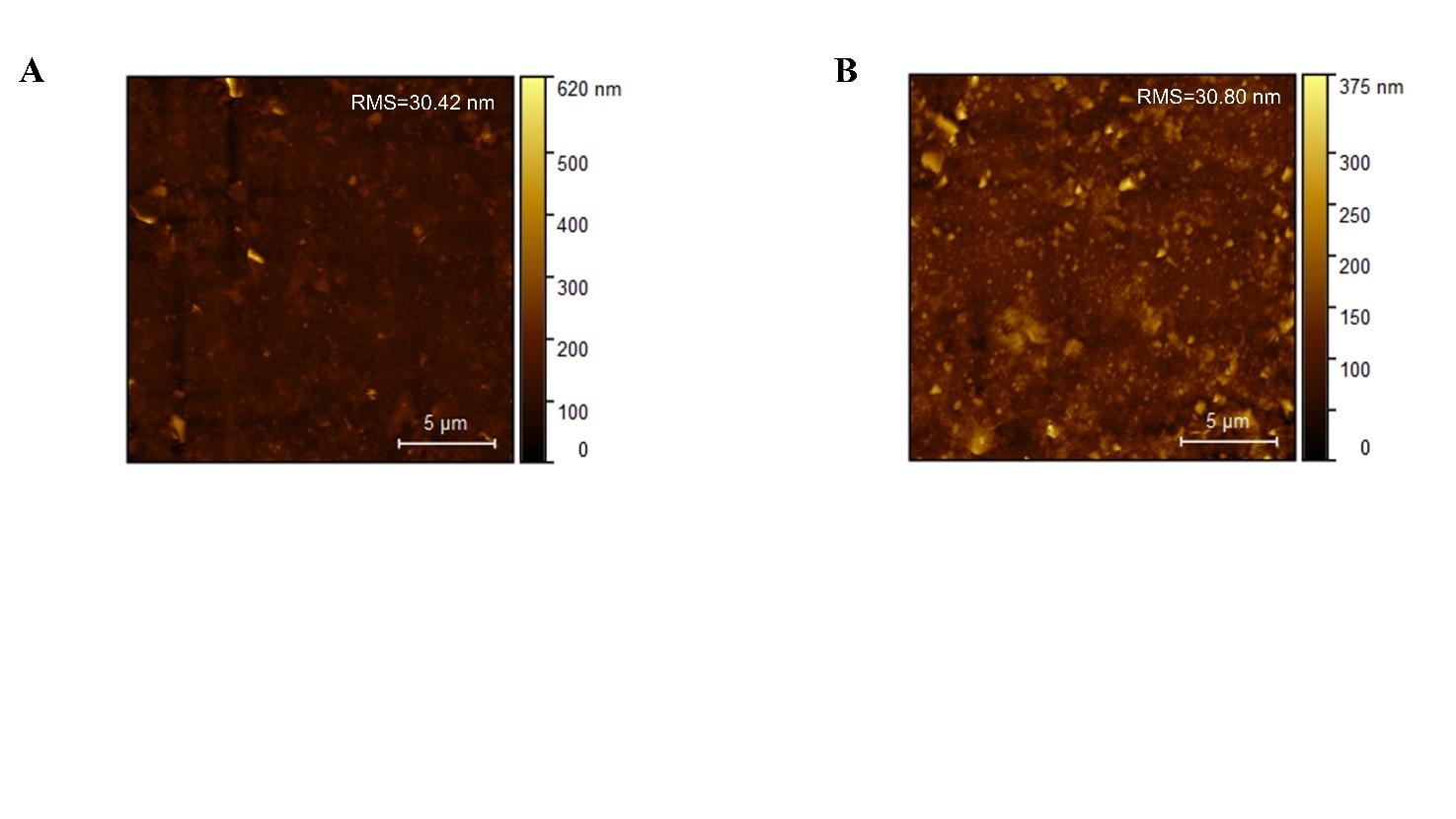
**

**Figure S12.** Surface topography images of EEG films prepared by spray-coating A) 5 ml and B) 10 ml of EEG(TCPP) (1 mg/ml) aqueous dispersion. Included are the RMS values.


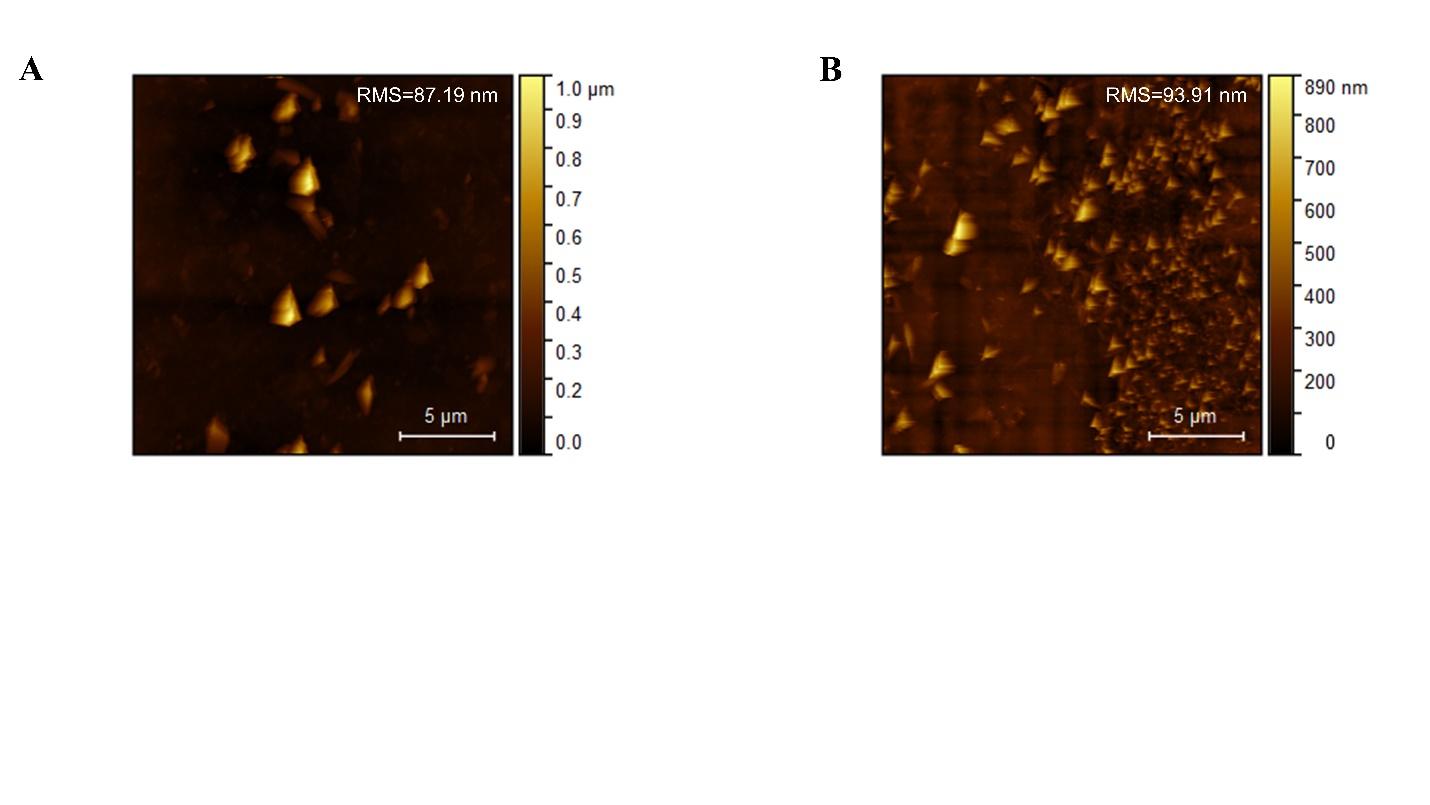


**Figure S13.** Surface topography images of EEG films prepared by spray-coating A) 5 ml and B) 10 ml of EEG(Sn-TCPP) (2.5 mg/ml) aqueous dispersion. Included are the RMS values.


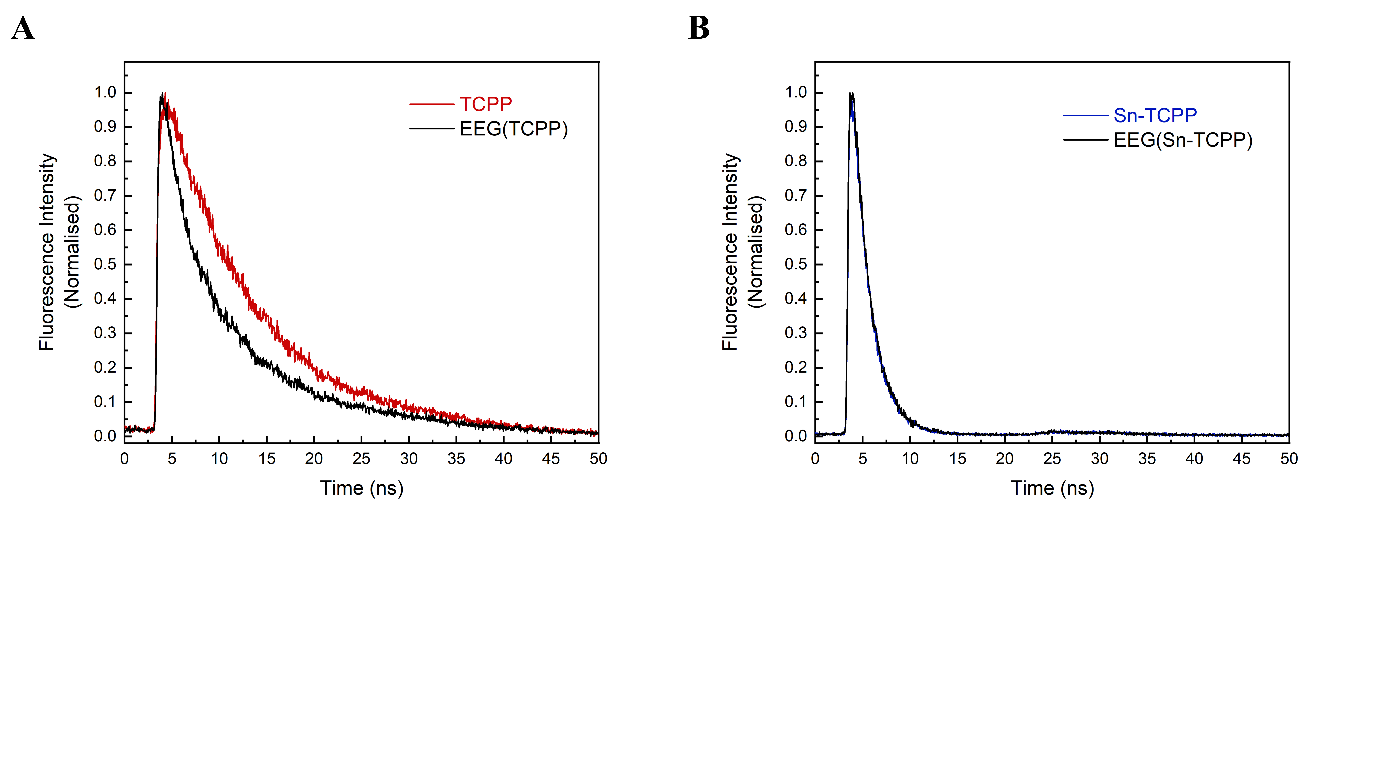


**Figure S14.** Fluorescence lifetime profiles of the photoexcited A) EEG(TCPP) and B) EEG(Sn-TCPP samples, with the respective photoexcited porphyrins as references.
